# L-glutamine Induces Expression of *Listeria monocytogenes* Virulence Genes

**DOI:** 10.1371/journal.ppat.1006161

**Published:** 2017-01-23

**Authors:** Adi Haber, Sivan Friedman, Lior Lobel, Tamar Burg-Golani, Nadejda Sigal, Jessica Rose, Nurit Livnat-Levanon, Oded Lewinson, Anat A. Herskovits

**Affiliations:** 1 Department of Biochemistry, The Bruce and Ruth Rappaport Faculty of Medicine, the Rappaport Institute for Biomedical research, Technion—Israel Institute of Technology, Haifa, Israel; 2 The Department of Molecular Microbiology and Biotechnology, The George S. Wise Faculty of Life Sciences, Tel Aviv University, Tel Aviv, Israel; McGill University Health Centre, CANADA

## Abstract

The high environmental adaptability of bacteria is contingent upon their ability to sense changes in their surroundings. Bacterial pathogen entry into host poses an abrupt and dramatic environmental change, during which successful pathogens gauge multiple parameters that signal host localization. The facultative human pathogen *Listeria monocytogenes* flourishes in soil, water and food, and in ~50 different animals, and serves as a model for intracellular infection. *L*. *monocytogenes* identifies host entry by sensing both physical (*e*.*g*., temperature) and chemical (*e*.*g*., metabolite concentrations) factors. We report here that L-glutamine, an abundant nitrogen source in host serum and cells, serves as an environmental indicator and inducer of virulence gene expression. In contrast, ammonia, which is the most abundant nitrogen source in soil and water, fully supports growth, but fails to activate virulence gene transcription. We demonstrate that induction of virulence genes only occurs when the *Listerial* intracellular concentration of L-glutamine crosses a certain threshold, acting as an on/off switch: off when L-glutamine concentrations are below the threshold, and fully on when the threshold is crossed. To turn on the switch, L-glutamine must be present, and the L-glutamine high affinity ABC transporter, GlnPQ, must be active. Inactivation of GlnPQ led to complete arrest of L-glutamine uptake, reduced type I interferon response in infected macrophages, dramatic reduction in expression of virulence genes, and attenuated virulence in a mouse infection model. These results may explain observations made with other pathogens correlating nitrogen metabolism and virulence, and suggest that gauging of L-glutamine as a means of ascertaining host localization may be a general mechanism.

## Author Summary

To thrive in the human body and cause disease, bacterial pathogens rely on the expression of a battery of genes, termed virulence genes. However, to activate these genes, the bacterium must “know” that it is no longer free-roaming, and has invaded a host. Like all life forms, bacterial pathogens must acquire the four elemental building blocks (carbon, nitrogen, phosphate, sulfur). The sources for these building blocks in the bacterial host are different from those present in the environment. It therefore makes perfect “biological sense” for bacteria to use such nutrients as localization beacons. We show for the first time, that the human pathogen *Listeria monocytogenes* surveys the available nitrogen sources before turning on its virulence genes. L-glutamine, an abundant nitrogen source in humans, greatly induces the expression of all major virulence genes. In contrast, a nitrogen source that is prevalent in soil and water, such as ammonia, fails to do so. To better treat infectious diseases, it is vital that we understand how bacteria switch to their virulent, disease-causing state. Our results provide a significant step in this direction.

## Introduction

*Listeria monocytogenes* is a Gram-positive facultative intracellular bacterial pathogen and the causative agent of listeriosis in humans, a disease with deleterious impacts, such as increased risk of meningitis and miscarriage [1]. *L*. *monocytogenes* invades mammalian cells by expressing surface proteins named internalins, that bind host proteins to induce active bacterial uptake [2]. Upon entry, *L*. *monocytogenes* escapes the vacuole (phagosome) by producing a pore-forming hemolysin, listeriolysin O (LLO, encoded by the *hly* gene) and two additional phospholipases, PlcA and PlcB [3], [4]. Once in the host cytosol, *L*. *monocytogenes* multiplies rapidly and expresses ActA, which recruits the host actin polymerization machinery to propel the bacteria within the cytosol and facilitate its spread from cell to cell [5]. Most of the known virulence factors involved in internalization, vacuolar escape and cell-to-cell spread are positively regulated by PrfA, the master virulence activator of *L*. *monocytogenes* [6], [7].

Immediately upon infection, *L*. *monocytogenes* senses multiple host-derived signals that alert the bacteria of their intracellular localization. Temperature, iron, and the availability of specific metabolites control the transcription, translation, and activity of PrfA and consequently, the induction of virulence genes. For example, carbon sources that are encountered by *L*. *monocytogenes* in the soil (*e*.*g*., glucose and cellobiose), have been shown to repress PrfA activity [8]. In contrast, carbon sources encountered in the host (*e*.*g*., glucose-1-phosphate and glycerol) are positive regulators of *prfA* [9]–[11]. The metabolism and virulence of *L*. *monocytogenes* are intimately linked via the combined regulation of PrfA and the global metabolism regulator CodY [12], [13]. The latter, among other things, senses low concentrations of branched-chain amino acids (BCAAs—isoleucine, leucine, and valine), as encountered within mammalian cells, and as a result affects transcription of *prfA* [14]. Recently, host derived glutathione was shown to serve as yet another intracellular signal that activates PrfA via allosteric binding [15], [16]. Together, these and other signals reflect the metabolic conditions within the mammalian cell and assist the bacteria in apprising the required metabolic and virulence adaptations.

While *L*. *monocytogenes* is well equipped to sense the mammalian niche, the host cell also employs sophisticated mechanisms to detect and respond to invading pathogens. For example, macrophage cells respond to *L*. *monocytogenes* infection by a robust activation of the type I interferon response, manifested by expression and secretion of the interferon β (IFN-β) and interleukin-6 (IL-6) [17], [18]. This response requires replication of *L*. *monocytogenes* within the host cell cytosol and secretion of c-di-AMP and other nucleic acids, demonstrating the existence of a specific sensing machinery for metabolically active bacteria [19], [20]. These findings establish that both *L*. *monocytogenes* and its host have evolved techniques to sense the metabolic state of each other and respond accordingly.

In efforts to identify *L*. *monocytogenes* novel genes that participate in host sensing and contribute to virulence, we have recently employed an *L*. *monocytogenes* transposon mutant library to infect bone marrow derived macrophages [21]. Using a reporter cell line featuring type I interferon-dependent luciferase expression [22], we screened approximately 5,000 *L*. *monocytogenes* mutants for decreased induction of IFN-β response. Among the identified mutants were several well-established virulence determinants of *L*. *monocytogenes* (*e*.*g*., PrfA), as well as newly identified potential virulence factors [21]. One of these genes, *LMRG_02270*, which encodes a polypeptide that is a fusion of a substrate-binding protein (SBP) and a transmembrane domain (TMD) of an ATP Binding Cassette (ABC) transporter, was chosen for further analysis. ABC transporters comprise a large super-family of proteins that couple the energy of ATP hydrolysis to the translocation of molecules across biological membranes against their concentration gradient [23–25]. ABC transporters have been shown to be involved in bacterial virulence and pathogenesis, as they allow the bacteria to acquire essential nutrients from the host [26–32].

In this work, we analyze the function of the *L*. *monocytogenes* ABC transporter GlnPQ, and show its importance in interpreting a newly identified signal that induces the expression of virulence genes: L-glutamine.

## Results

### Deletion of *LMRG_02270–1* genes leads to a reduced type I interferon response in macrophage cells

**Fig 1:**
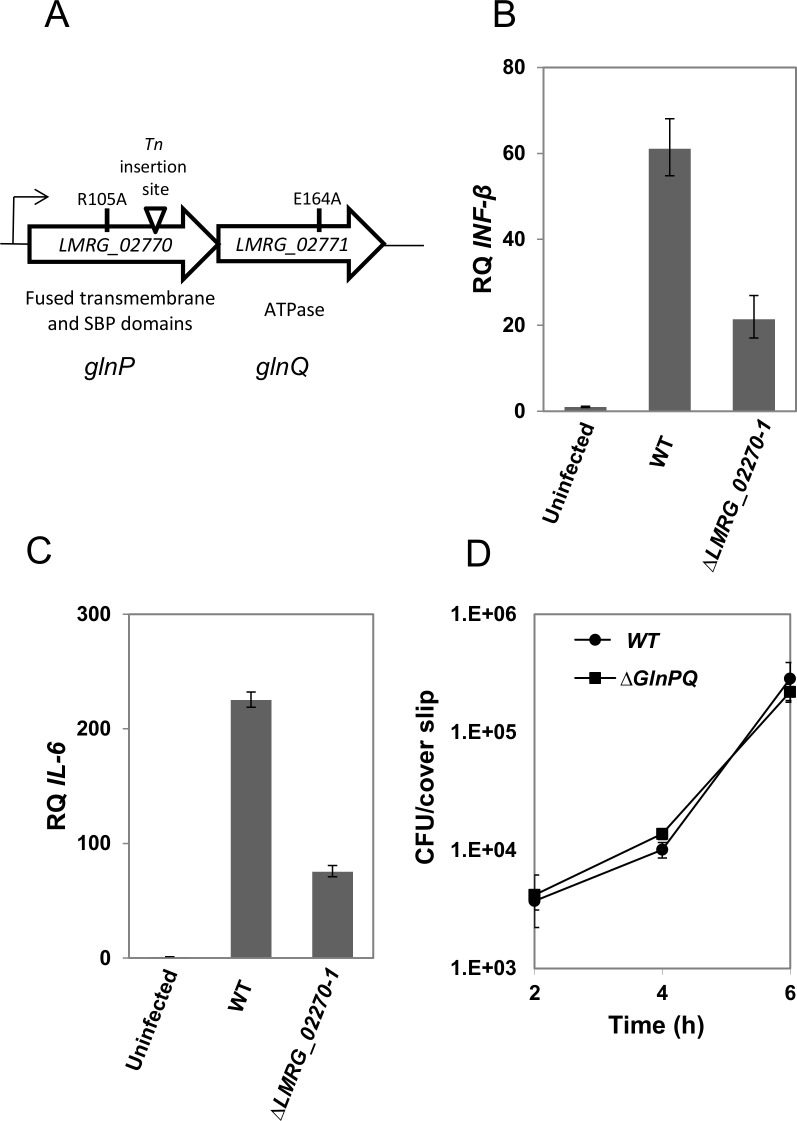
The *LMRG_02270–1* operon is required for activation of the Type I interferon response during macrophage cell infection. (A) Schematic representation of the *LMRG_02270–1* operon, which contains fused transmembrane and substrate binding protein (SBP) domains and an ATPase that together form the complete transporter. The transposon insertion site and the E164A and R105A mutations are indicated. RT-qPCR analysis of transcription of IFN-β (B) or IL-6 (C) in BMDMs 6 h post infection with WT *L*. *monocytogenes* (WT) or Δ*LMRG_02270–1* mutant, are indicated. Transcription levels are represented as relative quantity (RQ), relative to uninfected cells. Data represents at least 3 biological repeats. Error bars represent 95% confidence interval. (D) Intracellular growth curves, described in Colony Forming Units (CFU), of WT *L*. *monocytogenes* (circles) or Δ*LMRG_02270–1* mutant (squares) in BMDM cells. Representative growth curves of 3 biological repeats are shown. Error bars represent standard deviation of triplicates.

*LMRG_02270* is the first gene of a two-gene operon (Fig 1A), the second of which, *LMRG_02271*, contains all of the canonical motifs of a nucleotide-binding domain (NBD) of an ABC transporter (S1 Fig). Since both genes together are predicted to consist a functional ABC transport system, a mutant bearing deletions of both *LMRG_02270* and *LMRG_02271* (*LMRG_02270–1* double mutant) was prepared and tested for induction of type I interferons response in infected bone marrow derived macrophages (BMDMs). To this end, total mRNA from macrophages infected with either the *LMRG_02270–1* mutant or wild-type (WT) *L*. *monocytogenes* was extracted and IFN-β and IL-6 transcription levels were quantified 6 hours post-infection, using real-time quantitative PCR (RT-qPCR). Indeed, the *LMRG_02270–1* mutant elicited a much weaker type I interferon response in comparison to WT bacteria (Fig 1B and 1C), despite identical replication rates between the two bacterial populations (Fig 1D). Collectively, these results demonstrate that *LMRG_02770–1* contributes to the induction of the innate Type I interferon immune response to intracellularly growing *L*. *monocytogenes* bacteria.

### *LMRG_02270–1* operon encodes an ABC importer of L-glutamine

In ABC transporters that function as importers, the SBP is located extracellularly, where it recognizes the substrate with high affinity and delivers it to the transmembrane domain. A BLAST analysis of LMRG_02770 against the *E*. *coli* K-12 genome, revealed the highest homology to several ABC transporters (importers) of charged/polar amino acid, including those of glutamate, aspartate, glutamine, histidine, arginine and lysine.

**Fig 2:**
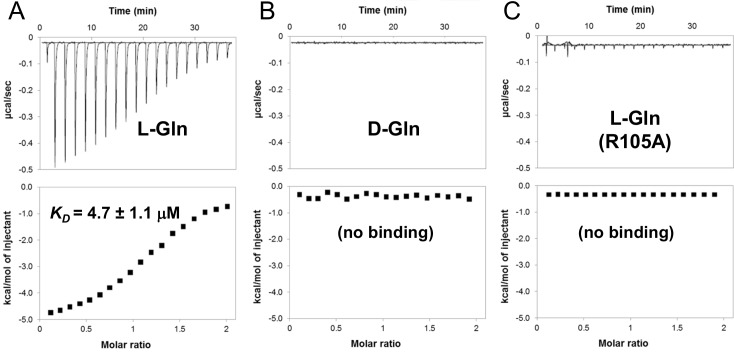
The SBP domain of LMRG_02270 specifically binds L-glutamine. Isothermal titration calorimetry was used to determine the binding of (A) L-glutamine or (B) D-glutamine to the SBP domain, or (C) of L-glutamine to the R105A mutant of the SBP domain. Shown are the consecutive injections of 2 μL aliquots from a 500 μM solution of the indicated amino acid, into 200 μL of 50 μM SBP. The upper panels show the calorimetric titration and the lower panels display the integrated injection heat derived from the titrations, for which the best-fit curve was used to calculate the *K*_*D*_. The experiments were conducted five times, and the *K*_*D*_ value is mean ±SD of 5 independent experiments.

The transport specificity of an ABC importer is almost exclusively determined by the binding specificity of the SBP [33–35]. Therefore, to identify the substrate specificity of the LMRG_02270–1 transporter, a truncated, His-tagged version of the LMRG_02770 SBP domain (containing only amino acids 29–254, *i*.*e*. without the transmembrane domain) was cloned, then overexpressed in *E*. *coli* and purified to near homogeneity (S2 Fig). Isothermal titration calorimetry (ITC) was then applied to determine the binding specificity of the SBP to different amino acids. Of the tested amino acids (L-isomers of Gln, Glu, Asn, Asp, His, Arg, Lys, Cys, Ser, Thr, and Tyr), detectable binding was only observed with L-glutamine, with a dissociation constant (*K*_*D*_) of 4.7 μM (Fig 2A). To increase the fraction of protonated glutamate we repeated the experiments at pH 6.5 (relative to the initial pH 8), yet still detected no binding. The SBP was also highly stereo-specific as no interaction was detected with D-glutamine (Fig 2B).

Using the published X-ray structure of the *Enterococcus faecalis* L-glutamine SBP (PDB 4G4P), we constructed a structural homology model of the SBP domain of LMRG_02270. Similar to its homologues, the model of the SBP domain showed the characteristic positioning of the conserved amino acid residues that constitute the substrate-binding site in glutamine binding proteins (S3 Fig). A number of these residues were conserved not only in glutamine binding proteins [36], but also in SBPs of histidine and arginine transporters [37]. Of these, the highly conserved Arg residue (R105 in LMRG_02270) is responsible for coordinating the backbone carboxy group of the amino acid. Thus, an arginine to alanine (R105A) mutation was designed, and indeed found to fully abolish L-glutamine binding (Fig 2C). Collectively, these results suggest that LMRG_02270 and LMRG_02271 form a high-affinity L-glutamine-specific import system in *L*. *monocytogenes*. In accordance with the nomenclature used in other bacteria [36], [38], the gene encoding the SBP-TMD (*LMRG_02770*) was designated *glnP*, and the gene encoding the ATPase (*LMRG_02271*) was designated *glnQ*.

### GlnPQ is the only high-affinity L-glutamine import system in *L*. *monocytogenes*

**Fig 3:**
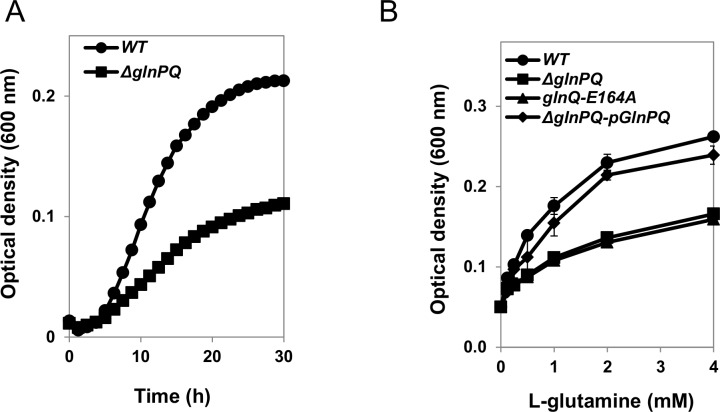
GlnPQ is required for L-glutamine utilization by *L*. *monocytogenes*. (A) Growth of WT *L*. *monocytogenes* (WT circles) or Δ*glnPQ* (squares) in MDM supplemented with 1 mM L-glutamine as the sole nitrogen source. Shown are representative growth curves of 3 independent experiments, performed in triplicates. (B) Optical density (600 nm) measurements after 30 h of growth of WT *L*. *monocytogenes* (circles), Δ*glnPQ* (squares), Δ*glnPQ*-pGlnPQ (diamonds), or *glnQ*-E164A (triangles) bacteria in MDM supplemented with the indicated concentrations of L-glutamine as the sole nitrogen source. Results are mean ±SD of 3 independent experiments, performed in triplicates.

Like most organisms, *L*. *monocytogenes* is unable to metabolize atmospheric nitrogen, and must acquire it in an organic form. Since L-glutamine was shown to be utilized by *L*. *monocytogenes* as a major nitrogen source [39], we hypothesized that a *ΔglnPQ* mutant strain would show attenuated growth when supplied with glutamine as a sole nitrogen source. When grown in rich media (brain-heart infusion (BHI)) the growth of the *ΔglnPQ* and WT strains was very similar (S4 Fig), indicating uncompromised overall fitness of this strain. In contrast, when the *ΔglnPQ* strain was grown on minimal defined medium (MDM) containing 1 mM L-glutamine as the sole nitrogen source, it displayed a 2-fold lower growth rate and a 2-times lower bacterial count in the stationary phase, relative to the WT bacteria (Fig 3A). Even at very high L-glutamine concentrations, the *ΔglnPQ* mutant failed to grow to the same extent as the WT strain (Figs 3B and S5). On the other hand, a *ΔglnPQ* strain complemented with *glnPQ* genes delivered on the pPL2 plasmid (*ΔglnPQ*-pGlnPQ), displayed the same growth profile as WT bacteria (Fig 3B). To verify that L-glutamine utilization requires active transport by GlnPQ, the conserved Walker B glutamic acid (E164), that is essential for ATP hydrolysis and transport activity [33], [34], [40], was mutated to alanine. The *glnQ*-E164A mutant failed to restore L-glutamine utilization, and its growth was indistinguishable from that of the *ΔglnPQ* strain (Fig 3B). Taken together, these results indicate high dependency of *L*. *monocytogenes* on the GlnPQ transporter for acquisition of L-glutamine under limiting conditions.

**Fig 4:**
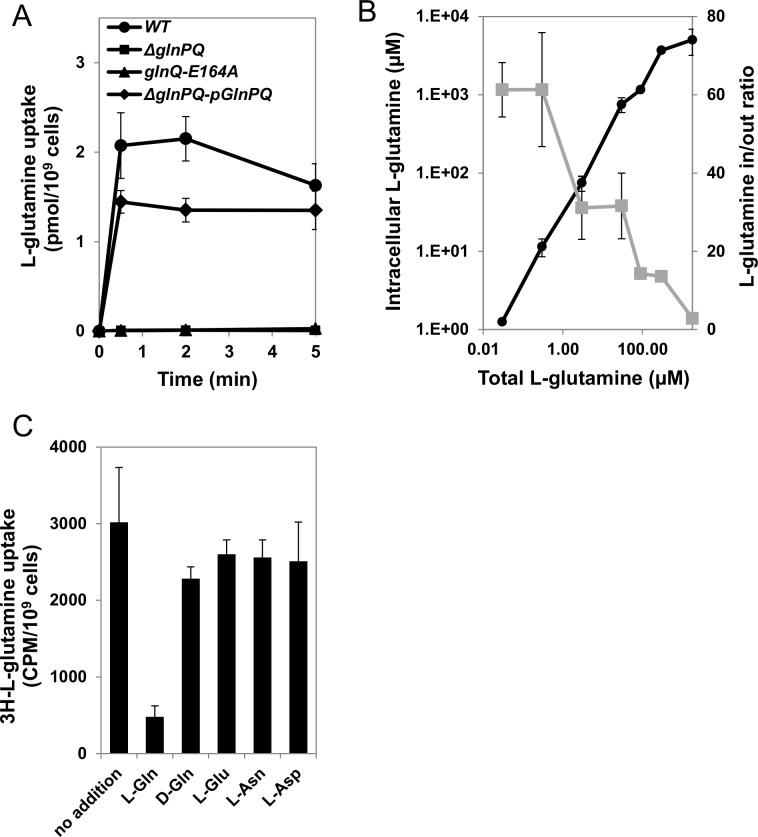
L-glutamine uptake by GlnPQ is rapid and selective. (A) 30 nM of ^3^H L-glutamine were added to WT (circles), Δ*glnPQ* (squares), Δ*glnPQ*-pGlnPQ (diamonds) or *glnQ*-E164A (triangles) bacteria. At the indicated time-points total L-glutamine uptake was measured by the rapid filtration method. (B) The intracellular concentration (black, left axis), and the concentration gradient (C_in_/C_out_) (grey, right axis) of L-glutamine were determined for WT bacteria at the indicated concentration of external L-glutamine. (C) Uptake of ^3^H-labeled L-glutamine (in counts per minute) by WT bacteria incubated for 5 min with ^3^H L-glutamine (3 μM) and in the absence or presence of 900 μM of the indicated competing amino acids. In C, shown is only the amount of accumulated label, not total L-glutamine. Results are mean ±SD of 3 independent experiments performed in triplicates.

This dependency was further demonstrated by comparing the uptake rates of ^3^H-labeled L-glutamine by WT versus *ΔglnPQ* bacteria. L-glutamine uptake by WT bacteria reached saturation within 30 sec, which was shorter than the temporal resolution of our assay. This rapid plateau of accumulation was observed over a broad range (0.03–3 μM) of L-glutamine concentrations (Fig 4A).

In contrast, the *ΔglnPQ* and *glnQ*-E164A mutant strains displayed practically zero uptake of L-glutamine (Fig 4A), even at concentrations as high as 300 μM, whereas the GlnPQ-complemented strain displayed the same rapid L-glutamine uptake as the WT strain (Fig 4A). These findings confirm that the rapid uptake of L-glutamine measured in the transport experiments is indeed mediated by an active, ATPase-dependent transport process, in which GlnPQ serves as the only high-affinity glutamine import system in *L*. *monocytogenes*. This conclusion is supported by a BLAST analysis of the genome of *L*. *monocytogenes* that failed to identify additional (putative) glutamine importers. Similarly, a bioinformatics study [41] revealed that unlike other Gram positive bacteria, *L*. *monocytogenes* harbors a single putative transporter in their nitrogen regulon, which is *glnPQ*.

Next, we measured the concentration gradients (Gln_in_:Gln_out_) generated by GlnPQ over a broad range of external glutamine concentrations (0.03–1800 μM L-glutamine). Of note, the determination of concentration gradients assumes that L-glutamine is not further metabolized intracellularly. However, even within the short time scale of the transport experiments this is only an approximation. Therefore, the calculated concentration gradients are to be viewed only as estimates. We observed that GlnPQ generated the greatest concentration gradients (Gln_in_:Gln_out_ of ~ 60) when the external concentration of L-glutamine was < 1 μM. For example, when L-glutamine was added externally at 30 nM, its internal concentration reached ~ 2 μM (Fig 4B). At higher external concentrations of L-glutamine the concentrative ability of GlnPQ gradually decreased: at 200 μM (external) L-glutamine the Gln_in_:Gln_out_ ratio decreased to ~10, leading to an internal concentration of ~ 2 mM, and a modest 2–3-fold concentration gradient was formed at 1 mM of externally added L-glutamine (Fig 4B). In agreement with the strict binding specificity of the SBP domain (Fig 2), addition of a 300-fold excess of unlabeled L-Asp, L-Asn, L-Glu, or D-Gln did not affect the amount of ^3^H L-glutamine that was taken up. Only the addition of unlabeled L-glutamine significantly reduced the amount of accumulated radioactive label (Fig 4C). These results substantiate the high specificity of GlnPQ for L-glutamine, with little to no cross-reactivity with other amino acids.

### The *ΔglnPQ* mutant displays reduced transcription of virulence genes

**Fig 5:**
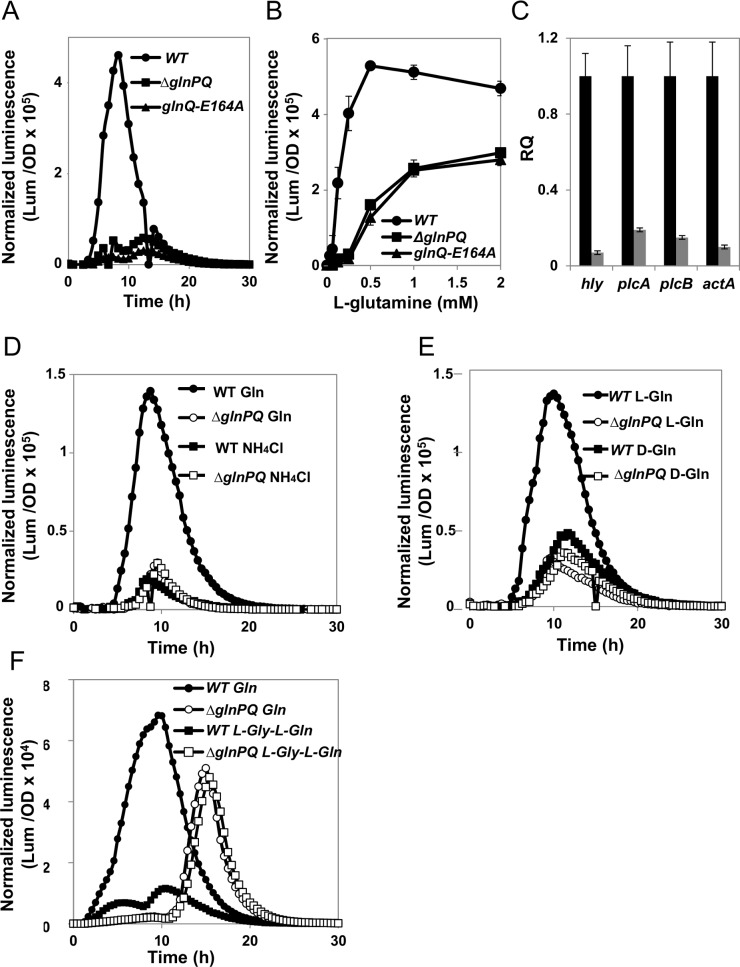
Virulence genes transcription is reduced in the *ΔglnPQ* mutant and specifically depends on L-glutamine. (A) Time course measurements of normalized luminescence (Lum/OD) indicative of *hly* promoter activity in WT *L*. *monocytogenes* (circles), Δ*glnPQ* (squares) or *glnQ*-E164A (triangles) bacteria grown in MDM with 0.25 mM of L-glutamine. Shown are representative curves of 3 independent experiments, performed in triplicates. (B) Maximal normalized luminescence (Lum/OD) was measured for WT (circles), Δ*glnPQ* (squares) or *glnQ*-E164A (triangles) bacteria grown under the indicated concentrations of L-glutamine. Results are mean ±SD of 3 independent experiments performed in triplicates. (C) RT-qPCR analysis of *hly*, *plcA*, *plcB*, and *actA* transcript levels in WT *L*. *monocytogenes* (black bars) or Δ*glnPQ* (gray bars) bacteria grown in MDM with 4 mM L-glutamine. Transcription levels presented as relative quantity (RQ), relative to their levels in WT bacteria. Results are mean ±SD of 3 independent experiments performed in triplicates. (D) Time course measurements of normalized luminescence (Lum/OD) indicative of *hly* promoter activity for WT (circles) and *ΔglnPQ* (squares) bacteria grown on MDM with 0.5 mM L-glutamine (full symbols) or 0.5 mM NH_4_Cl (empty symbols) as the sole nitrogen source. (E) Time course measurements of normalized luminescence (Lum/OD) indicative of *hly* promoter activity for WT (circles) and Δ*glnPQ* (squares) bacteria grown on MDM with 0.5 mM L-glutamine (full symbols) or 0.5 mM D-glutamine (empty symbols) as the sole nitrogen source. (F) Time course measurements of normalized luminescence (Lum/OD) indicative of *hly* promoter activity for WT (circles) and Δ*glnPQ* (squares) bacteria grown on MDM with 0.5 mM L-glutamine (full symbols) or 0.5 mM L-Gly-L-Gln dipeptide (empty symbols) as the sole nitrogen source.

To further study the role of GlnPQ in *L*. *monocytogenes* virulence, we first examined the transcription of the *hly* gene, encoding for Listeriolysin O toxin (LLO), a well-established *L*. *monocytogenes* virulence factor, essential for phagosomal escape [3]. To this end, the pPL2 integrative plasmid harboring the *hly* promoter fused to a luminescent *luxABCDE* reporter system (pPL2 *hly-lux*) [42], was conjugated to WT, Δ*glnPQ* and *glnQ*-E164A bacteria. Luminescence, indicative of *hly* transcription, was recorded during 30 hours of growth in MDM supplemented with L-glutamine (0.25 mM) as a sole nitrogen source. Under these conditions, a high and sharp luminescence peak was observed in WT bacteria, reaching maximal levels in the middle of the exponential growth phase. In contrast, the clean deletion and the point mutation strains almost completely failed to express the *lux* genes, indicating no activation of the *hly* promotor (Fig 5A).

Luminescence was then quantified over a broad range of L-glutamine concentrations (0.02–2 mM). Relative to the *ΔglnPQ* and *glnQ*-E164A mutants, WT bacteria consistently presented a much higher activity of the *hly* promoter, reaching half its maximal activity at about 100–200 μM L-glutamine. At these external L-glutamine concentrations the *ΔglnPQ* and *glnQ*-E164A mutants displayed almost zero induction of *hly* (Fig 5B). In complementary RT-qPCR experiments, we similarly observed a dramatic (~20-fold) reduction of *hly* mRNA transcripts in *ΔglnPQ* versus WT bacteria (Fig 5C). The *ΔglnPQ* mutant also exhibited a 5–10-fold reduction in transcription of other major *Listerial* virulence factors such as: *plcA*, *plcB* and *actA* (Fig 5C).

Collectively, these results demonstrate that an active GlnPQ transporter is important for transcription of *L*. *monocytogenes* virulence genes.

### Activation of virulence genes specifically depends on the presence of L-glutamine yet is unrelated to nitrogen availability

One possible interpretation of the reduced transcription of the virulence genes in the *ΔglnPQ* strain is that nitrogen starvation restrains their expression. To test this, we sought to satisfy the metabolic need for nitrogen by supplying a nitrogen source other than L-glutamine.

**Fig 6:**
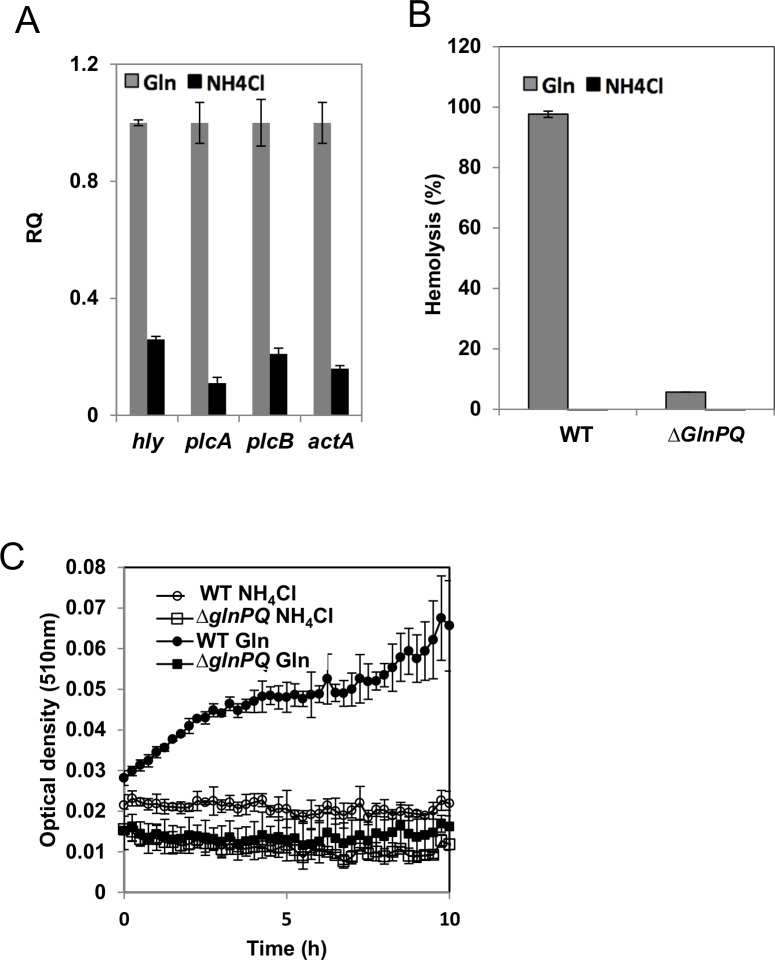
The transcription and activity of virulence genes depends on L-glutamine. (A) RT-qPCR transcription analysis of *hly*, *plcA*, *plcB*, and *actA* in WT bacteria grown in MDM with either 4 mM L-glutamine (gray bars) or 4 mM NH_4_Cl (black bars) as a sole nitrogen source. Transcription levels are presented as relative quantity (RQ), relative to levels in WT. Results are mean ±SD of 3 independent experiments performed in triplicates. (B) Hemolysis activity of LLO in culture supernatants of WT and *ΔglnPQ* bacteria grown on minimal defined media with 1 mM L-glutamine (grey) or 4 mM NH_4_Cl (black) as the sole nitrogen source. (C) Analysis of PI-PLC activity of PlcA in culture supernatants of WT (circles) and *ΔglnPQ* (squares) bacteria grown on minimal defined media with 1 mM L-glutamine (full symbols) or 4 mM NH_4_Cl (empty symbols) as the sole nitrogen source. Representative graphs of 3 biological repeats are shown. Error bars represent standard deviation of the triplicate.

As semi-complex nitrogen sources we used a chemically defined media to which we added a tryptone or a peptone peptide digest. For growth in the presence of a defined nitrogen source we used a chemically defined media supplemented with ammonia, arginine, glutamate, or D-glutamine. As previously reported [39], [43], [44], *L*. *monocytogenes* grew well when supplied with ammonia as the sole nitrogen source (S6A Fig). However, despite the normal growth, luminescence driven from the *hly* promoter was very low in both WT and *ΔglnPQ* mutant, similar to those observed in the *ΔglnPQ* mutant grown in the presence of L-glutamine (Fig 5D). Similar results were obtained using tryptone or a peptone peptide digest as the sole nitrogen source: WT and the *ΔglnPQ* bacteria strain grew equally well (S6B Fig), yet in both cases the luminescence was very low (S6C and S6D Fig). This indicates that the *ΔglnPQ* strain can still take up and utilize nitrogen from short peptides. Unlike short peptides or ammonia, glutamate and arginine proved to be poor nitrogen sources, and could not support growth even when supplied at high (10–20 mM) concentrations. *L*. *monocytogenes* could also use D-glutamine as a nitrogen source, and in line with the strict stereo-specificity of GlnPQ (Fig 2B), the *ΔglnPQ* mutant was not impaired in its ability to use D-glutamine (S6E Fig). As observed with ammonia or short peptides for both the WT and the *ΔglnPQ* mutant *hly* transcription was not activated when the nitrogen source was D-glutamine (Fig 5E). Collectively, these results show that nitrogen starvation *per se* is not the limiting factor for virulence machinery activation, and that L-glutamine is specifically required for the expression of the virulence genes. This conclusion is strongly supported by experiments where nitrogen was supplied in the form of a synthetic L-Gly-L-Gln dipetide. WT and *ΔglnPQ* bacteria efficiently utilized this di-peptide (S6F Fig), and displayed comparable *hly*-associated luminescence, that was nearly as high as the luminescence observed in the presence of L-glutamine (Fig 5F). The dependence of virulence genes transcription on the identity of the nitrogen source was also studied in RT-qPCR experiments. As shown, despite the normal growth (S6A Fig), the transcription level of all examined virulence genes (*hly*, *plcA*, *plcB* and *actA*) was lower in the presence of ammonia relative to their transcription levels in the presence L-glutamine (Fig 6A). In accordance, no protein activity of LLO (encoded by the *hly* gene) and PlcA was detected in culture supernatants of WT bacteria grown in the presence of ammonia in comparison to L-glutamine, as well as in supernatants of *ΔglnPQ* bacteria (Fig 6B and 6C). Since activation of the virulence genes depends on the master virulence regulator PrfA, we examined whether L-glutamine affects its transcript or protein level. For this, WT and *ΔglnPQ* bacteria were grown in the presence of L-glutamine or ammonia and PrfA’s mRNA and protein levels were measured by RT-qPCR and Western blot analyses. As shown, a higher *prfA* transcription level was observed in WT bacteria grown with L-glutamine in comparison to ammonia and to *ΔglnPQ* bacteria (S7A Fig). This increased transcription level was expected as under virulence conditions *prfA* is also transcribed together with *plcA*, which was shown to be induced upon L-glutamine uptake (Fig 6A). Nevertheless, the Western blot analysis of PrfA protein demonstrated similar levels of PrfA under all conditions (S7B Fig). While these findings indicate that L-glutamine does not affect PrfA transcription or translation, it is still possible that PrfA is regulated by L-glutamine at the protein level.

Higher resolution inspection of the dependence of virulence gene transcription on L-glutamine levels revealed a non-linear and non-hyperbolic, on/off switch-type response (S8 Fig). A Hill coefficient of n_Hill_ = 2.73 was calculated for the glutamine dependence, indicative of high signal amplification; very low *hly* induction is observed at low L-glutamine concentrations, and very high *hly* induction is achieved once a threshold concentration is crossed. These data suggest that the on/off transition occurs at external L-glutamine concentrations of ~100–200 μM. At these external concentrations the pumping activity of GlnPQ brings internal L-glutamine to ~3 mM (Fig 4B, black curve, left Y-axis). For maximal growth, *L*. *monocytogenes* requires 2–4 mM external L-glutamine (Fig 3B). However, saturation of *hly* transcription occurred at 10–20-fold lower concentrations (S8 Fig). Therefore, full manifestation of the virulence gene induction occurs long before full satisfaction of the bacteria’s metabolic needs.

### GlnPQ contributes to *L*. *monocytogenes* virulence in bone marrow-derived macrophages and mice

**Fig 7:**
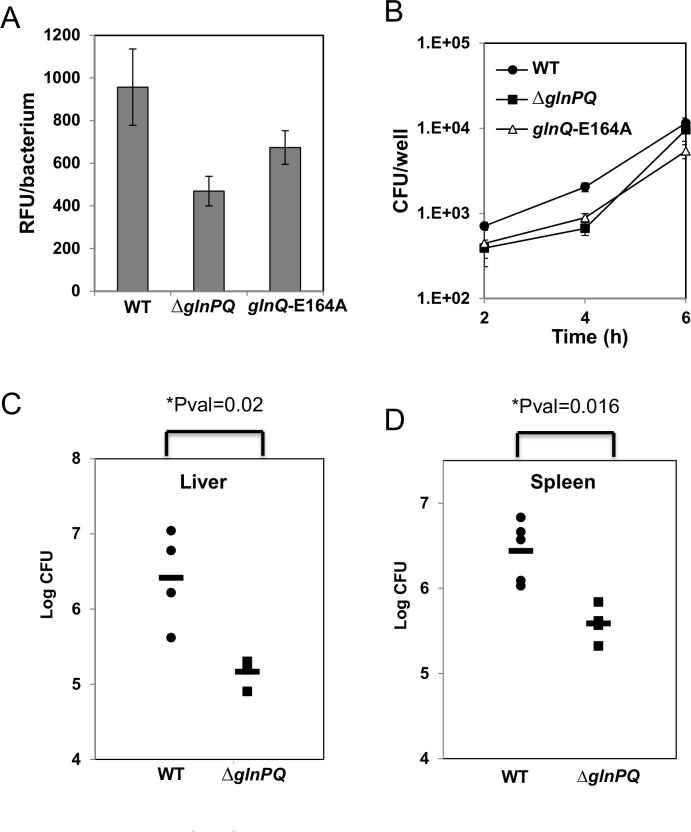
GlnPQ promotes *L*. *monocytogenes* virulence. (A) Intracellular detection of YFP fluorescence indicative of *plcA* promoter activity (pPL2-*plcA-yfp*) in *WT L*.*m*., Δ*glnPQ* and *glnQ*-E164A, at 3.5 hours post infection of BMDMs grown in glutamine-restricted medium (B) Intracellular growth of WT *L*. *monocytogenes* (circles), Δ*glnPQ* mutant (squares) and *glnQ*-E164A (triangles) in BMDM cells grown in glutamine-restricted medium. Representative growth curves of 3 biological repeats are shown. Error bars represent standard deviation of the triplicate. Bacterial counts in the spleens (C) and livers (D) of infected animals as analyzed at 72 h post infection. Mice were injected intravenously with 4 × 10^4^ WT *L*. *monocytogenes* (circles) or Δ*glnPQ* (squares) cells.

To assess the role of GlnPQ during *L*. *monocytogenes* infection of BMDMs, macrophage cells were grown in glutamine-restricted medium (no glutamine was added, and DMEM without glutamine was used), and then infected with WT, *ΔglnPQ* or *glnQ*-E164A bacteria. The transcription level of the virulence genes was assessed by measuring the transcription level of *plcA*, using a *plcA-yfp* transcriptional fusion (3 consecutive *yfp* genes were expressed from the integrative pPL2 plasmid under the regulation of the *plcA* promoter). We monitored YFP fluorescence, and observed reduced *plcA* expression in *ΔglnPQ* and *glnQ*-E164A bacteria in comparison to WT bacteria (Fig 7A). Accordingly, *ΔglnPQ* and *glnQ*-E164A bacteria displayed lower infectivity than WT, but only when the macrophages were grown in L-glutamine-restricted medium, relative to when they were grown in L-glutamine-enriched (4 mM) medium (Compare Fig 7B, restricted, to Fig 1C, enriched). These results suggest that L-glutamine sensing is important during early stages of infection.

Finally, to determine the contribution of GlnPQ to *L*. *monocytogenes* virulence in mice, young female C57BL/6 mice were intravenously injected with 4 × 10^4^ cells of the *ΔglnPQ* mutant or WT strain, and bacterial counts in the spleens and livers of infected mice were analyzed at 72 h post infection. As shown in Fig 7C and 7D, relative to the WT, the *ΔglnPQ* mutant colonized the livers and spleens to a lesser extent, exhibiting a 30-fold (liver) and a 10-fold (spleen) decrease in recovered bacteria. Collectively, these results clearly demonstrate that GlnPQ is necessary to promote *L*. *monocytogenes* virulence.

## Discussion

*L*. *monocytogenes* is a highly adaptive bacterium that successfully inhabits diverse environmental niches. It is routinely isolated from soil, silage, decaying plants, and water reservoirs. It is also known to infect at least 50 different animals, and to invade almost all types of mammalian cells. Clearly, each niche presents different challenges and therefore, to survive *L*. *monocytogenes* must constantly adjust its lifestyle. When the bacterium is free-roaming, the environments it traverses are usually spatially continuous (*e*.*g*., a water reservoir and its banks) and present overlapping metabolic requirements. From a temporal perspective, the environmental changes encountered by the freely roaming bacterium are likely to be gradual (*e*.*g*., temperature, pH, salinity). Yet, when entering a host, environmental changes are both spatially and temporally abrupt. The host’s environment is spatially isolated from the external environment, it presents very different metabolic requirements, and poses unique survival challenges mounted by the immune system. Therefore, a facultative pathogen such as *L*. *monocytogenes* must feature tools to rapidly and efficiently react to such conditions. Indeed, upon infection, *L*. *monocytogenes* activates elaborate survival machineries that power altered motility, metabolism, mammalian cell penetration, phagosomal escape and cell-to-cell spreading. However, to activate these machineries the bacterium must be able to sense that it is no longer free roaming, and is inside a host. Several such host-based cues have been previously identified, including temperature (37°C), pH, carbon source identity and availability, and specific metabolites such as branched-chain amino acids and glutathione [14], [15], [45–47]. As activation of the entire host-related survival machinery on the basis of a single cue would clearly be detrimental, the bacteria integrate multiple signals before switching to a virulent state. This work identified and characterized the role of L-glutamine levels in bacterial sensing of the host environment.

It appears that *L*. *monocytogenes* has only one system for the high affinity uptake of L-glutamine: GlnPQ. When GlnPQ is deleted, or inactivated, there is no detectable uptake of L-glutamine, and reduced growth in media that contains L-glutamine as the sole nitrogen source. Nevertheless, the *ΔglnPQ* strain can still grow if supplied with high concentrations of L-glutamine. This points to the existence of alternative, low affinity pathways for the uptake of L-glutamine. Importantly, the general fitness of the *ΔglnPQ* mutant is not impaired, as evidenced by its normal growth in rich media, or in medium supplemented with tryptone, peptone, ammonia, or D-glutamine as a nitrogen source.

Here we show that at external L-glutamine concentrations of 100–200 μM, the pumping activity of GlnPQ brings the intracellular concentration to ~3 millimolar. This does not happen in *ΔglnPQ* bacteria. Importantly, this influx of L-glutamine turns on the induction of virulence genes in WT bacteria. Consequently, the *ΔglnPQ* mutant shows reduced activation of BMDM cells, reduced expression of a battery of virulence genes, reduced infectivity in macrophage cells under conditions of L-glutamine restriction, and reduced virulence *in vivo*.

Nutrient starvation-driven repression of virulence gene expression has been reported for *Mycobacterium tuberculosis* in relation to phosphate starvation [48], [49]. However, this is clearly not the case here since alternative nitrogen sources, despite satisfying the bacteria’s growth needs, do not induce expression of virulence genes. It is also quite remarkable that even D-glutamine cannot replace the L isomer as a virulence activation signal. Collectively, the results demonstrate the heavy dependence of virulence gene activation specifically on L-glutamine, as well as absence of interconnection between the virulence gene activation and cellular nitrogen quota machineries.

### But how is external L-glutamine differentiated from one that is internally produced? How can L-glutamine be used as cue for host localization?

When WT bacteria are supplied with ammonia and glutamate they synthesize L-glutamine (via the glutamine synthase GlnA [39]). Similarly, *L*. *monocytogenes* successfully extracts glutamine from a complex mixture of peptides (peptone or tryptone digest). However, in all of these cases, despite the intracellular production of L-glutamine virulence genes are not induced. We think that the distinction between externally supplied and internally produced L-glutamine is achieved via a threshold-based “switch mechanism”. Transcription of virulence genes only starts when the internal concentration of L-glutamine crosses a threshold, which according to our data is ~3 mM. In the absence of external L-glutamine the internally synthesized molecules are incorporated into proteins, utilized in other anabolic processes, or catabolically consumed, never reaching the concentrations that are needed to cross the activation threshold. The threshold can only be crossed via the concentrative action of GlnPQ in WT bacteria that are fed with L-glutamine, or when the bacteria are fed with an L-glutamine containing dipeptide.

### What is the advantage of using L-glutamine as a signal for host localization?

In retrospect, it makes perfect “biological sense” to use L-glutamine as an environmental signal. Gaseous nitrogen (N_2_) is the most abundant molecule in the atmosphere (78%), but can only be exploited by a handful of organisms, primarily soil-residing, ‘nitrogen fixing’ bacteria that express the enzyme nitrogenase. In the soil, nitrogenase catalyzes the reduction of atmospheric nitrogen (N_2_) to ammonia (NH_3_), which is the most abundant nitrogen source in the soil. Amino acids in general, and L-glutamine specifically, are scarce. In stark contrast, ammonia concentrations in the serum and cells of mammals are very low, while those of L-glutamine are very high (200–500 μM to several mM) [50–53], making L-glutamine a perfect signal for host localization. In this regard it is important to note that within the cytosolic niche *L*. *monocytogenes* most likely utilizes multiple nitrogen sources, for example ethanolamine and possibly glucosamine, but whether they also serve as signals for virulence gene activation is currently not known [39], [54], [55]. Of note, we were unable to test whether ethanolamine can serve as yet another signal, since *L*. *monocytogenes* bacteria could not grow in minimal medium with it as a sole nitrogen source unless glycerol was added, which is itself an inducer of virulence gene expression [10].

While we identified and characterized the L-glutamine transport system, we do not know how L-glutamine is sensed by the bacteria and how the signal is transduced. We found that L-glutamine does not activate *prfA* transcription or translation, though it is still possible it activates PrfA at the protein level. Notably, the crystal structure of PrfA shows a snugly bound L-glutamine, even though it was not included in the crystallization mixture [56]. Interestingly, the binding site of L-glutamine overlaps with the one proposed for GSH, which was recently shown to be an allosteric activator of PrfA [15]. In addition to PrfA, GlnR, the global nitrogen regulator common to many bacteria, and GlnA (the glutamate synthase) may serve as sensors/regulators. Aside from its enzymatic function, GlnA regulates the transcription of the *glnRA* operon and is also required for the regulatory functions of GlnR [57]. GlnA activity is directly controlled by L-glutamine [58], [59], and therefore L-glutamine also indirectly controls the regulatory functions of GlnR. Importantly, both genes have been correlated with virulence in other pathogens [52], [60], [61]. In this regard, in *L*. *monocytogenes* the global metabolic regulator CodY, that activates transcription of the global virulence regulator PrfA, has been shown to regulate also GlnR [13]. A correlation between nitrogen metabolism and virulence was observed also for other bacterial pathogens, including *Staphylococcus aureus* [62], [63], *Streptococcus pneumonia* [29], [57], [60], *Salmonella typhi* [61], *Group B Streptococci* [64], and *Mycobacterium tuberculosis* [52]. However, it is unknown whether in these pathogens, virulence specifically depends on L-glutamine, or on nitrogen availability in general. The mechanism of L-glutamine sensing, and the generality of the mechanism across different pathogens, are open questions that remain to be addressed.

## Materials and Methods

### Ethics statement

Experimental protocols were approved by the Tel Aviv University Animal Care and Use Committee (01-15-052, 04-13-039) according to the Israel Welfare Law (1994) and the National Research Council guide (Guide for the Care and Use of Laboratory Animals 2010).

### Bacterial strains and growth media

*Listeria monocytogenes* 10403S was used as the WT strain and as the parental strain to generate allelic exchange mutant strains (S1 Table). *E*. *coli* XL-1 Blue strain (Stratagene) was used for generation of vectors and *E*. *coli* SM-10 strain [65] was used for plasmid conjugation to *L*. *monocytogenes*. *L*. *monocytogenes* strains were grown in BHI (Merck) rich medium, or in minimal defined medium (MDM) [66], [67]. For growth under limiting conditions, MDM without arginine was freshly prepared, supplemented with various concentrations of L-glutamine, NH_4_Cl, D-glutamine or L-Gly-L-Gln (Sigma).

### Generation of *L*. *monocytogenes* gene deletion strain, point mutation strains and complementation strains

All in-frame deletions and point mutation strains generated in this work were constructed using *L*. *monocytogenes* 10403S strain as the parental strain. Upstream and downstream regions of the selected gene were amplified by using Phusion DNA polymerase and cloned into the pKSV7oriT vector [68]. Cloned plasmids were sequenced and then conjugated to *L*. *monocytogenes* using the *E*. *coli* SM-10 strain. *L*. *monocytogenes* conjugants were selected on BHI–agar plates supplemented with chloramphenicol and streptomycin (10 μg/ml and 100 μg/ml, respectively), then grown at 41°C for two days on BHI–agar plates supplemented with chloramphenicol alone for plasmid integration into the bacterial chromosome by homologous recombination. For plasmid curing, bacteria were passed several times in fresh BHI without chloramphenicol at 30°C to allow plasmid excision by the generation of an in-frame deletion. Bacteria were then plated on BHI plates and chloramphenicol sensitive colonies were picked for validation of gene deletion by PCR using upstream and downstream primers. Complemented strains of deletion mutants were generated by introducing a copy of the deleted gene *in trans* under the control of its native promoter using the pPL2 integrative vector.

### Intracellular growth of *L*. *monocytogenes*

For all infection experiments *L*. *monocytogenes* strains were grown overnight in BHI medium at 30°C without shaking. Bone Marrow Derived Macrophages (BMDMs) used for infection experiments were isolated from 6–8 weeks-old female C57/BL6 mice (Harlan Laboratories, Israel) as described previously [69]. BMDMs were cultured in Dulbecco’s Modified Eagle Medium (DMEM)- based media supplemented with 20% fetal bovine serum, 1 mM sodium pyruvate, 2 mM L-glutamine, 0.05 mM β- Mercaptoethanol, monocyte colony stimulating factor (M-CSF) (L929-conditioned medium), and penicillin and streptomycin (5 μg ml^−1^ each). The cultures were incubated in a 37°C incubator with 5% CO_2_. 1×10^5^ macrophage cells per well in 100 μl of medium were seeded in a 96 well plate overnight. Before infection, macrophages cells were washed twice with phosphate buffer saline (PBS), and fresh medium without antibiotics was added. Approximately 1.6×10^3^ *L*. *monocytogenes* bacteria resuspended in PBS were used to infect each well. An hour post-infection, macrophage monolayers were washed twice with PBS and fresh medium supplemented with gentamicin (50 μg ml^−1^) was added to limit bacterial extracellular growth. At each time point, the media was aspirated from the wells and 100 μl of sterile water were added, followed by vigorous pipetting to release intracellular bacteria. Then serial dilutions of the lysate were plated on BHI-agar plates and Colony Forming Units (CFU) were counted after 24 h incubation at 37°C.

### Macrophage gene expression analysis

The transcriptional levels of macrophage genes were analyzed using RT-qPCR. RNA from infected macrophages was extracted using TRIzol reagent according to standard protocols (Biolab). 1 μg of RNA was reverse transcribed to cDNA using a QScript reverse transcription kit (Quanta). RT-qPCR was performed on 10 ng of cDNA using SYBR green with the StepOnePlus RT-PCR system (Applied Biosystems) (see S2 Table for RT-qPCR primers). The transcription levels of macrophage cytokine genes were normalized using glyceraldehyde-3-phosphate dehydrogenase (GAPDH) as a reference gene, and the uninfected cells as a reference sample.

### Cloning, overexpression and purification of GlnPQ SBP

The gene encoding the SBP portion of GlnPQ from *Listeria monocytogenes* 10403S (first 254 amino acids of LMRG_02770), but without its N-terminal signal sequence (first 28 amino acids), was synthesized and adjusted to the *E*. *coli* codon usage (Genescript). The gene was cloned into pET-19b (Novagen) vector for expression with an N-terminal His-tag [70], [71]. His-tagged GlnPQ SBP was overexpressed in *E*.*coli* BL21-Gold (DE3) cells (Stratagene) cultured in Terrific Broth (TB) and induced at mid log phase by addition of 1 mM Isopropyl b-D-1-thiogalactopyranoside (IPTG) for 1.5 h at 37°C. Cells were harvested by centrifugation (13,600 × *g*, 20 min, 4°C) and the pellet was stored at -80°C until use.

For purification, cells were homogenized in 50 mM Tris-HCl pH 8, 500 mM NaCl, complete EDTA-free protease inhibitor (Roche), 30 mg ml^-1^ DNase (Worthington), and 1 mM MgCl_2_. The cells were then ruptured by three passages in an EmulsiFlex-C3 homogenizer (Avestin), and the lysate centrifuged at 34,500 × *g* for 30 min at 4°C. The supernatant was loaded onto a nickel affinity column (HisTrap HP, GE Healthcare) on an AKTA Avant instrument. The protein was eluted using an imidazole gradient, and imidazole was eliminated from the sample by desalting (HiPrep 26/10, GE Healthcare). Protein purification was monitored by coomassie staining of SDS-PAGE and size exclusion chromatography (Superdex 75 10/300 GL, GE Healthcare).

The R105A point mutation was introduced to the GlnPQ SBP by the QuikChange Lightning site directed mutagenesis kit (Agilent Technologies) and confirmed by sequence analysis. The mutant protein was overexpressed and purified as described above for the wild type GlnPQ SBP.

### Isothermal titration calorimetry experiments

Calorimetric measurements were performed with Microcal iTC200 System (GE Healthcare). Prior to measurement, the protein was dialyzed against three exchanges of 50 mM Tris-HCl pH 8, 500 mM NaCl buffer. Amino acid stocks were prepared fresh in double-distilled water and diluted to working concentration using the buffer from the last protein dialysis exchange. 2 μL aliquots of 500 μM amino acid were added by a rotating syringe to the reaction well containing 200 μL of 50 μM WT or mutant GlnPQ SBP at 25°C [28]. Data fitting was performed with the Microcal analysis software.

### In vitro growth and luminescence of *L*. *monocytogenes* in laboratory media

For luminescence assays, *L*. *monocytogenes* strains harboring the P_*hly*_-luciferase reporter system (pPL2-P_*hly*_*lux*) were used. Bacteria from overnight cultures grown in BHI medium were adjusted to OD_600_ of 0.05 in fresh BHI or MDM containing various concentrations of L- glutamine, D-glutamine, NH_4_Cl or tryptone digest, and 150 μL were transferred into a clear flat bottom white 96-well plate. The plate was incubated in an InfiniteM200 pro (Tecan) at 37°C for a period of 12–40 hours, during which luminescence and OD_600_ were measured.

### L-glutamine transport experiments

Bacteria from overnight cultures grown in BHI medium were adjusted to OD_600_ of 0.05 in fresh BHI and grown at 37°C to an OD_600_ of 0.4. The cultures were harvested at 4°C, washed in ice-cold standard phosphate-buffer saline (pH 7.4) supplemented with 1.6 mM MgSO_4_, re-suspended to an OD_600_ of 15 and kept on ice until further analysis. Then, glucose (1% w/v) was added to a 50 μl aliquot, and the cells were allowed to recover for 10 min at 37°C. The transport assay was initiated by the addition of 2 μl of a solution containing the desired concentration of non-labelled L-glutamine and 0.1 μCi of L-[2,3,4-^3^H] glutamine (60 Ci/mmol) (American Radiolabeled Chemicals, Inc.). For competition assays, the solution contained also the competing amino acid to a working concentration of 900 μM. After incubation at 37°C for the indicated times, 3 ml of ice-cold assay buffer were added and the sample was immediately filtered through a moistened 2.4-cm diameter glass microfiber filter (GF/C; Whatman). The filter was then washed with an additional 3 ml of ice-cold assay buffer, dried and placed in a scintillation vial. 3 ml of Ultima Gold F scintillation fluid (Perkin Elmer) was added, and radioactivity was measured the following day on a β-counter.

### Calculations of intracellular glutamine concentrations

For all calculations of internal concentrations and concentration gradients we assumed that 100% of the accumulated label reports on glutamine, and that it has not been further metabolized during the transport experiment. The radioactive counts of several known concentrations of ^3^H-Gln were determined, including the stock solution that was later used in the uptake experiment. This was compared to the amount of radioactivity that was taken up by the cells. This amount was converted to concentrations assuming a cellular amount of 5·10^8^ cells per 1mL of OD 1 and a cellular volume of 1 μm^3^.

### Bacterial gene expression analysis

For RNA extraction, bacteria from overnight cultures were adjusted to OD_600_ of 0.05 in 20 ml of fresh MDM containing 4 mM of L-glutamine or NH_4_Cl as the sole nitrogen source. The cultures were incubated with agitation at 37°C to an OD_600_ of 0.3–0.4. Bacteria were harvested by centrifugation, washed with ice-cold PBS and stored frozen at -80°C until further analysis.

Total nucleic acids were extracted by the RiboPure RNA Purification Kit (Ambion). 1 μg of purified RNA was reverse transcribed to cDNA using the high-capacity cDNA reverse transcription kit (Applied Biosystems). RT-qPCR was performed on 10 ng of cDNA using PowerUp SYBR green master mix (Applied Biosystems) and 500 nM forward and reverse primers, designed with the Primer3web software (version 4.0.0) in the Rotor-Gene 6000 (Qiagen) system. The transcription level of each gene of interest was normalized to that of a reference gene, *rpoD*. Statistical analysis was performed using the Rotor-Gene Q series software. RT-qPCR primers are described in S2 Table.

### Measurements of LLO and PlcA proteins activity

LLO hemolytic activity assay was performed as described previously [46], [72]. Bacterial supernatants were treated with 5 mM DTT, serially diluted in PBS and incubated at 37°C with 0.5% of sheep blood red cells suspension (NovaMed Ltd.) in 35m M sodium phosphate buffer pH = 5.5, 125 mM NaCl, 0.5 mg/ml BSA. Cells were then removed by centrifugation at 1000 g and hemolysis was estimated as optical density at 541 nm. PlcA PI-PLC activity assay was adopted from [73], 0.05 gr phosphatidyl-inositol (Sigma P6636) were mixed by sonication with 10 ml of 0.2% sodium deoxycholate, 1 mM CaCl_2_, 114 mM (NH_4_)SO_4_ and 40 mM Tris-HCl pH = 7.2. 100μl of the assay solution was then mixed with 100 μl of bacterial supernatant and incubated in plate reader at 37C for 10 h, with continuous detection of turbidity at 510 nm.

### Western blot analysis

*Lm* bacteria were grown in MDM containing 4 mM of L-glutamine or ammonia as the sole nitrogen source at 37°C and harvested at a 0.3–0.4 OD. Then the bacteria were lysed by incubation with 5 units of mutanolysin (M9901; Sigma) for 1 hour at 37°C, followed by sonication. Cell debris were removed by centrifugation at 3000 g. Protein concentrations were quantified by modified Lowry method [74]. Samples with equal amounts of total proteins were separated on 12.5% SDS-PAGE, electro-blotted and probed either with rabbit anti-PrfA antibodies (made in this study, Almog Diagnostics, dilution 1:2500), followed by HRP-conjugated goat anti-rabbit IgG (Jackson ImmunoResearch, USA), respectively. Anti-GroEL antibodies (a kind gift from A. Azem, Tel Aviv University) were used as a loading control at a dilution of 1:20,000, followed by HRP-conjugated goat anti-rabbit IgG. Western blots were developed by enhanced chemiluminescence reaction (ECL).

### Intracellular *plcA* gene expression in BMDMs

WT and mutant strains of *L*. *monocytogenes* expressing three consecutive YFP proteins under the regulation of the *plcA* gene promoter in a pPL2 integrative plasmid were used to infect BMDMs on 20 mm slides. Upon infection, cells were cultured in BMDM medium with no added glutamine (traces of glutamine may exist due to addition of SCF to the medium). Four hours post infection, cells were fixed with 4% v/v paraformaldehyde-PBS and permeabilized with 0.05% v/v Triton X-100. DNA was stained with DAPI containing Vectashield mounting media (Vector laboratories inc.). Images were taken using Zeiss LSM 510-META confocal microscope.

### In vivo mice infections

*L*. *monocytogenes* bacteria were grown in BHI medium at 30°C overnight without shaking. Bacterial cultures were washed twice with PBS. BALB/c (6–8 weeks old) female mice (Harlan Laboratories) were infected via tail vein injections with 2.7×10^4^ bacteria in 200 μl PBS. Each group consisted of 5 mice for every mutant tested. Animals were observed daily for any signs of illnesses and were euthanized 72 h post-infection. Spleens and livers were harvested and homogenized in 0.2% v/v saponin. The numbers of viable bacteria in each organ were determined by plating serial dilutions of homogenates onto BHI agar plates.

## Supporting Information

S1 Fig Amino acid sequence and conserved motifs of *LMRG_02271* (the ATPase). The conserved ATP-binding cassette (ABC) motifs are color-coded and indicated.
(TIFF)

S2 Fig Purification of the SBP domain of *LMRG_02770*. (A) Coomassie staining of SDS-PAGE of Ni-NTA affinity purification of His-SBP. Lane 1: Total protein extract. Lane 2: Column-unbound fraction. Lanes 3–5: Eluted fractions with 0, 25 and 60 mM of imidazole, respectively. Lanes 6–11: fractions eluted using a linear gradient of 60 to 250 mM imidazole. Lane 12: The final product after pooling fractions 6–11. (B) Analysis of the purified protein by size exclusion chromatography. 20 μl of a 4 mg ml^-1^ protein solution were injected on a Superdex 75 10/300 GL column (GE Healthcare).
(TIFF)

S3 Fig Model structure of *LMRG_02270* SBP domain. A 3-D structural homology model was constructed for the SBP of *LMRG_02270* based on the X-ray structure of L-glutamine SBP from *Enterococcus faecalis* (PDB ID 4G4P) and using the Swiss-model software. Top: a side view of a cartoon representation (green), with L-glutamine shown as yellow sticks. Bottom: Zoom in of the binding site for L-glutamine (boxed). Conserved amino acids that participate in L-glutamine binding are shown as sticks and are labeled, as are relevant protein-substrate interactions. The Arg residue mutated in the R105A protein mutant is indicated by an arrow.
(TIFF)

S4 Fig The Δ*glnPQ* strain is not globally compromised. Growth curves of WT *L*. *monocytogenes* (circles) or Δ*glnPQ* (squares) bacteria grown in BHI broth. Representative curves are shown of 3 independent experiments performed in triplicates.
(TIFF)

S5 Fig Growth of WT *L*. *monocytogenes* and *ΔglnPQ* bacteria in minimal media supplemented with L-Gln as the sole nitrogen source. Growth curves of WT *L*. *monocytogenes* (black circles) or *ΔglnPQ* (gray squares) bacteria grown in minimal media containing 4, 2, 1, 0.5, 0.25, or 0.125 mM of L-Gln, as indicated. Representative curves are shown of 3 independent experiments performed in triplicates.
(TIFF)

S6 Fig Growth and *hly* luminescence in the presence of various nitrogen sources. (A) Optical density (600 nm) measurements after 30 h of growth of WT *L*. *monocytogenes* (circles) or *ΔglnPQ* (squares) bacteria in MDM supplemented with the indicated concentrations of Ammonium chloride as the sole nitrogen source. Results are mean ±SD of 3 independent experiments, performed in triplicates. (B) Optical density (600 nm) measurements after 30 h of growth of WT *L*. *monocytogenes* (circles) or *ΔglnPQ* (squares) bacteria in MDM supplemented with the indicated concentrations of tryptone (full symbols) or peptone (open symbols) peptide digest as the sole nitrogen source. Results are mean ±SD of 3 independent experiments, performed in triplicates. (C) Time course measurements of normalized luminescence (Lum/OD) indicative of *hly* promoter activity in WT *L*. *monocytogenes* (circles), *ΔglnPQ* (squares) or *glnQ*-E164A (triangles) bacteria grown in MDM with 0.5 mM of L-glutamine (full symbols) or a peptone (open symbols) peptide digest. Shown are representative curves of 3 independent experiments, performed in triplicates. (D) Same as C only 0.5 mM of L-glutamine (full symbols) or a tryptone (open symbols) peptide digest. (E) Optical density (600 nm) measurements after 30 h of growth of WT *L*. *monocytogenes* (circles) or *ΔglnPQ* (squares) bacteria in MDM supplemented with the indicated concentrations of D-Glutamine as the sole nitrogen source. Results are mean ±SD of 3 independent experiments, performed in triplicates. (F) Same as E, only the indicated concentrations of an L-Gly-L-Gln di-peptide were used as the sole nitrogen source.
(TIFF)

S7 Fig The effect of L-glutamine on *prfA* transcription and translation. WT *L*. *monocytogenes* and *ΔglnPQ* bacteria were grown in the presence of 4 mM of L-glutamine or NH_4_Cl and *prfA* transcript levels were measured using real-time q-PCR analysis (A), while PrfA protein levels were detected by Western blot analysis using an anti-PrfA antibody (B). Transcription levels are presented as relative quantity (RQ), relative to levels in WT bacteria grown with L-glutamine. Results are mean ±SD of 3 independent experiments performed in triplicates. The Western blot shown is representative of three independent biological repeats.
(TIFF)

S8 Fig Focusing on the transition between the ON and OFF states of *hly* transcription. Shown is the peak luminescence measurments (blue diamonds) of WT bacteria grown in MDM supplemented with the indicated concentrations of L-glutamine. The dashed line is the fit of the data using the Hill equation. Also given are the K_1/2_ and the Hill coefficient n_Hill_.
(TIF)

S1 Table Strains used in this study. (PDF)

S2 Table Oligonucleotides used in this study. (PDF)
