# Supplementary figures and images for: L-glutamine Induces Expression of Listeria monocytogenes Virulence Genes
Source: PLoS Pathog. 2017 Jan 23;13(1):e1006161. doi: 10.1371/journal.ppat.1006161 (PMC5289647; doi:10.1371/journal.ppat.1006161)

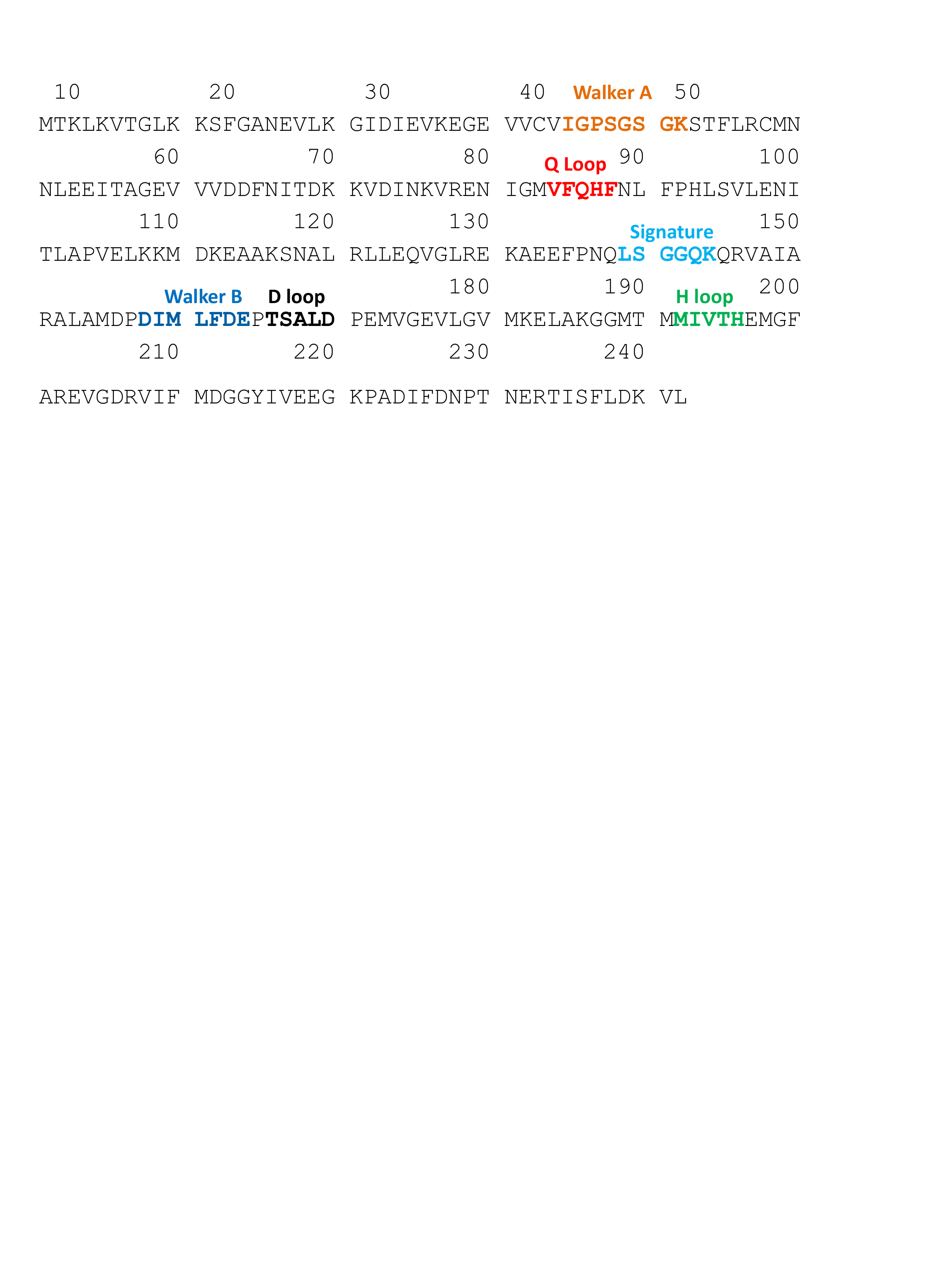

Supplement: S1 Fig — The conserved ATP-binding cassette (ABC) motifs are color-coded and indicated. (TIFF) [file ppat.1006161.s001.TIFF]

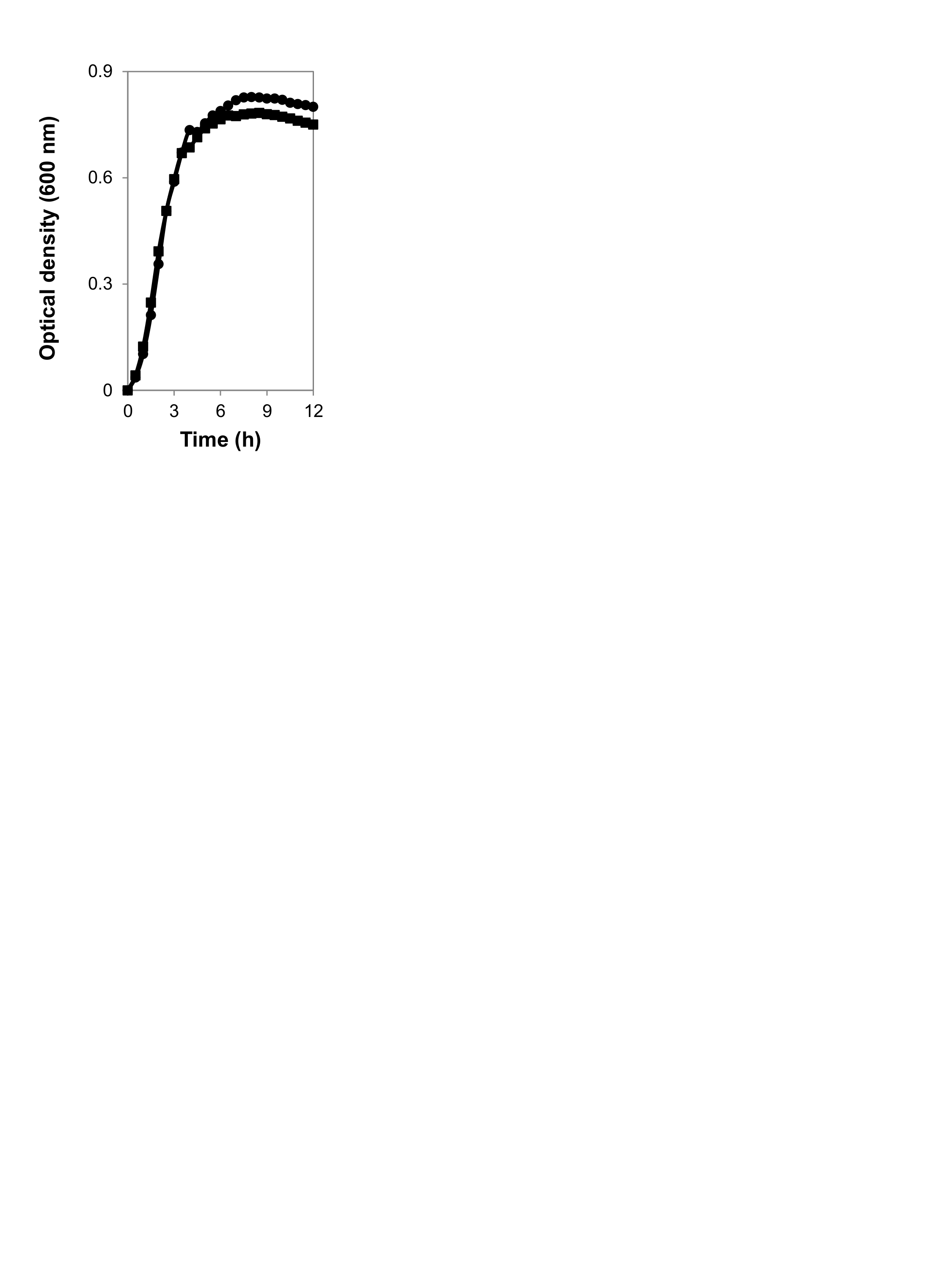

Supplement: S2 Fig — (A) Coomassie staining of SDS-PAGE of Ni-NTA affinity purification of His-SBP. Lane 1: Total protein extract. Lane 2: Column-unbound fraction. Lanes 3–5: Eluted fractions with 0, 25 and 60 mM of imidazole, respectively. Lanes 6–11: fractions eluted using a linear gradient of 60 to 250 mM imidazole. Lane 12: The final product after pooling fractions 6–11. (B) Analysis of the purified protein by size exclusion chromatography. 20 μl of a 4 mg ml-1 protein solution were injected on a Superdex 75 10/300 GL column (GE Healthcare). (TIFF) [file ppat.1006161.s002.TIFF]

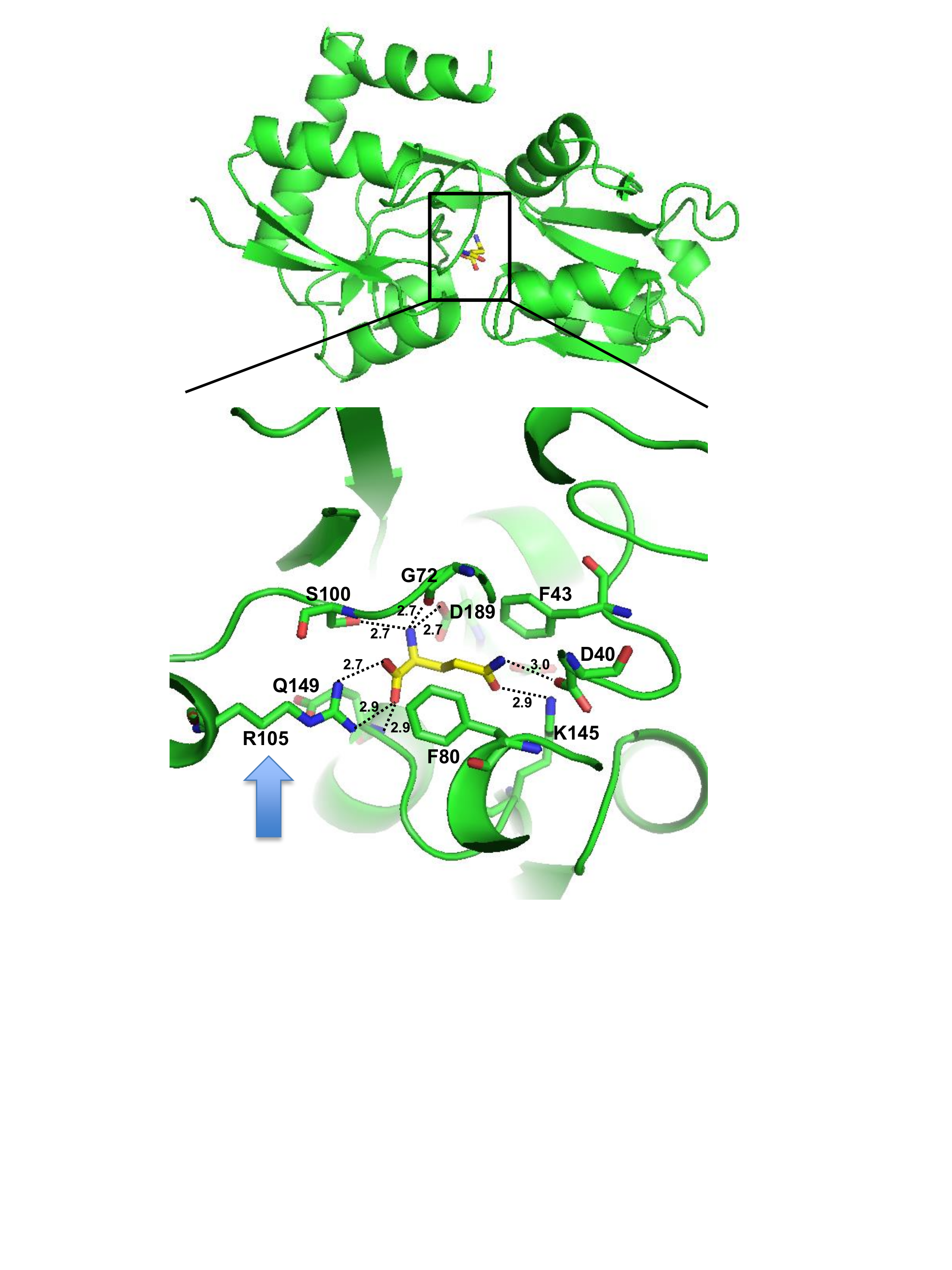

Supplement: S3 Fig — A 3-D structural homology model was constructed for the SBP of LMRG_02270 based on the X-ray structure of L-glutamine SBP from Enterococcus faecalis (PDB ID 4G4P) and using the Swiss-model software. Top: a side view of a cartoon representation (green), with L-glutamine shown as yellow sticks. Bottom: Zoom in of the binding site for L-glutamine (boxed). Conserved amino acids that participate in L-glutamine binding are shown as sticks and are labeled, as are relevant protein-substrate interactions. The Arg residue mutated in the R105A protein mutant is indicated by an arrow. (TIFF) [file ppat.1006161.s003.TIFF]

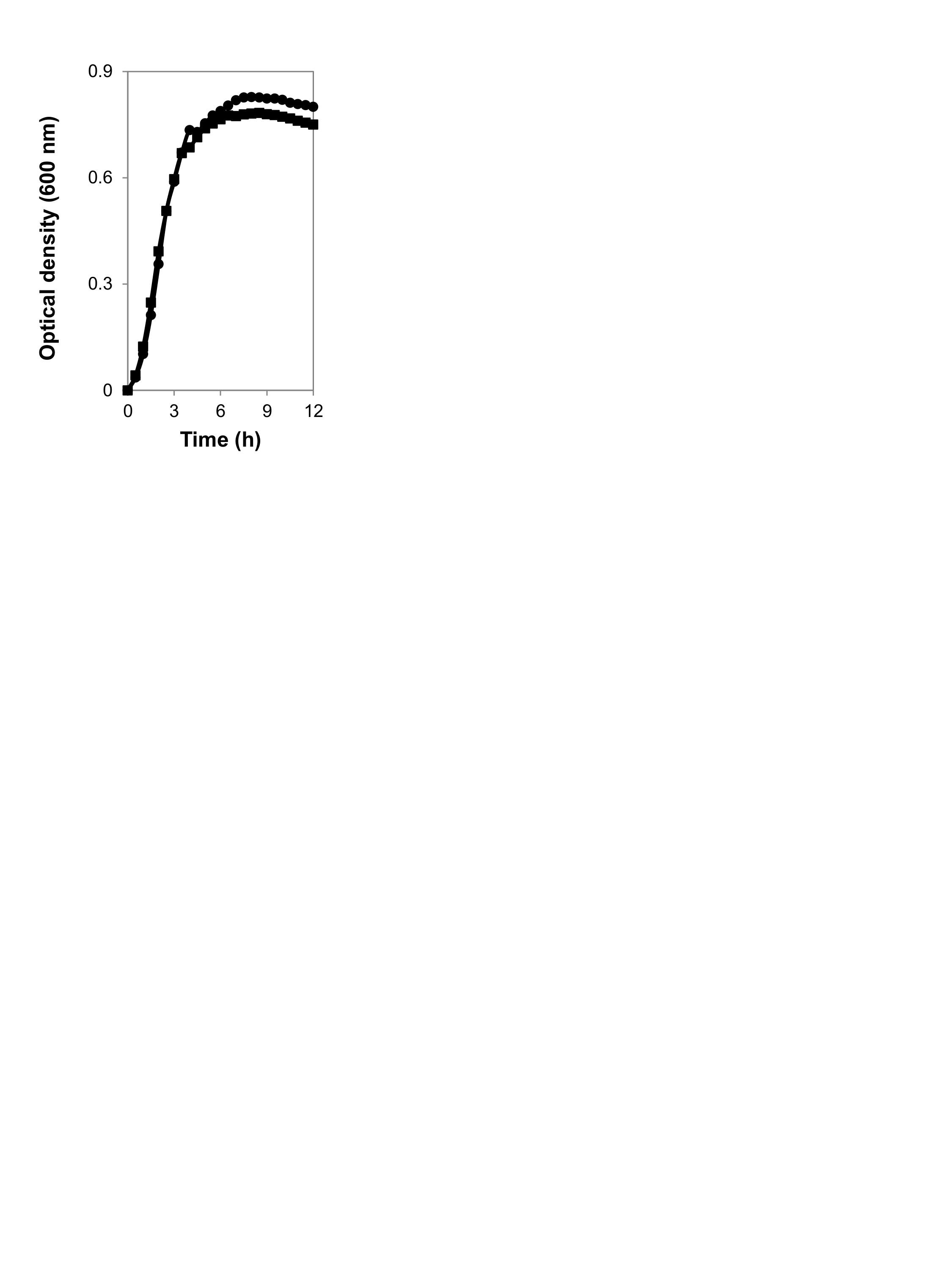

Supplement: S4 Fig — Growth curves of WT L. monocytogenes (circles) or ΔglnPQ (squares) bacteria grown in BHI broth. Representative curves are shown of 3 independent experiments performed in triplicates. (TIFF) [file ppat.1006161.s004.tiff]

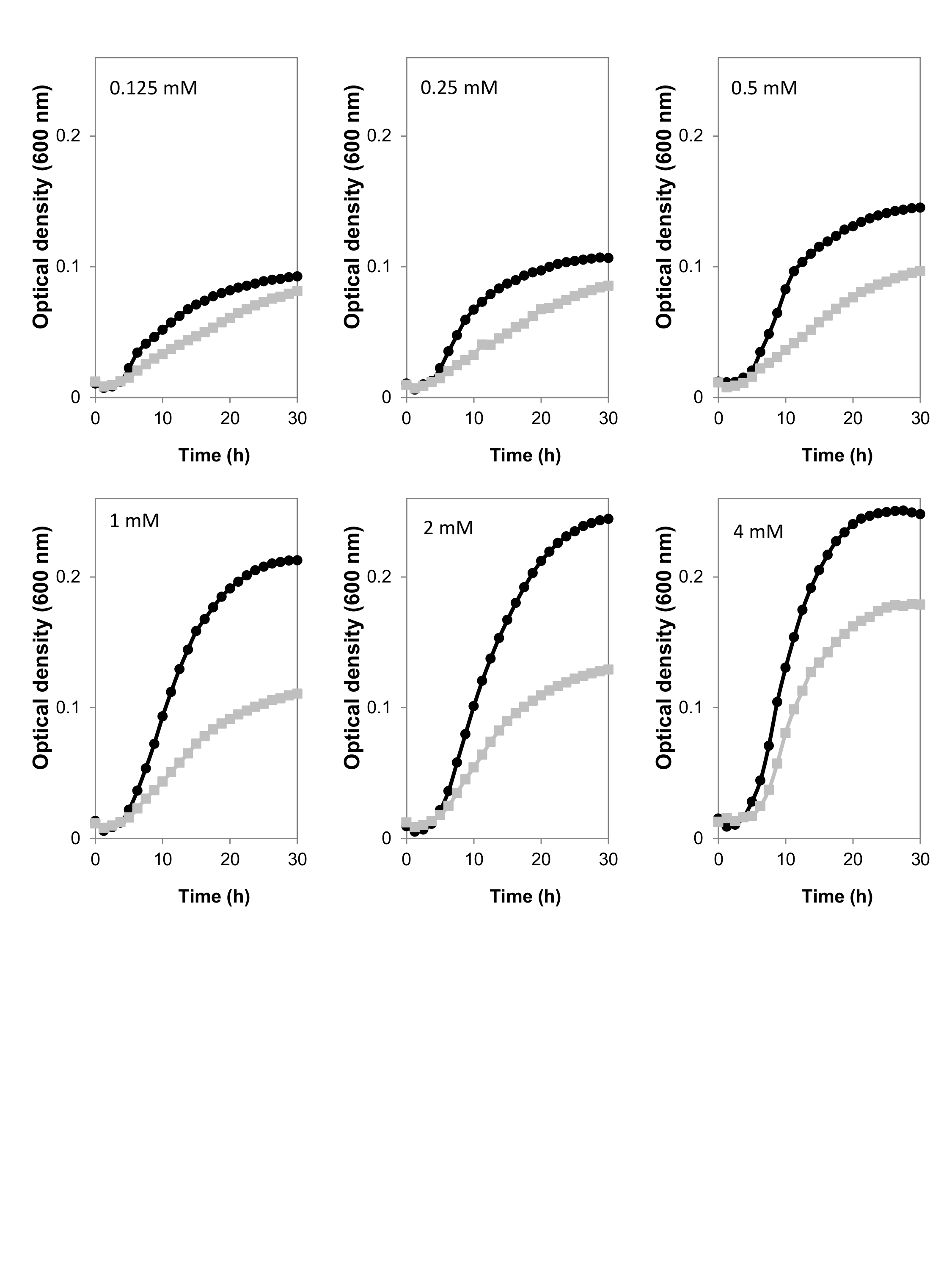

Supplement: S5 Fig — Growth curves of WT L. monocytogenes (black circles) or ΔglnPQ (gray squares) bacteria grown in minimal media containing 4, 2, 1, 0.5, 0.25, or 0.125 mM of L-Gln, as indicated. Representative curves are shown of 3 independent experiments performed in triplicates. (TIFF) [file ppat.1006161.s005.tiff]

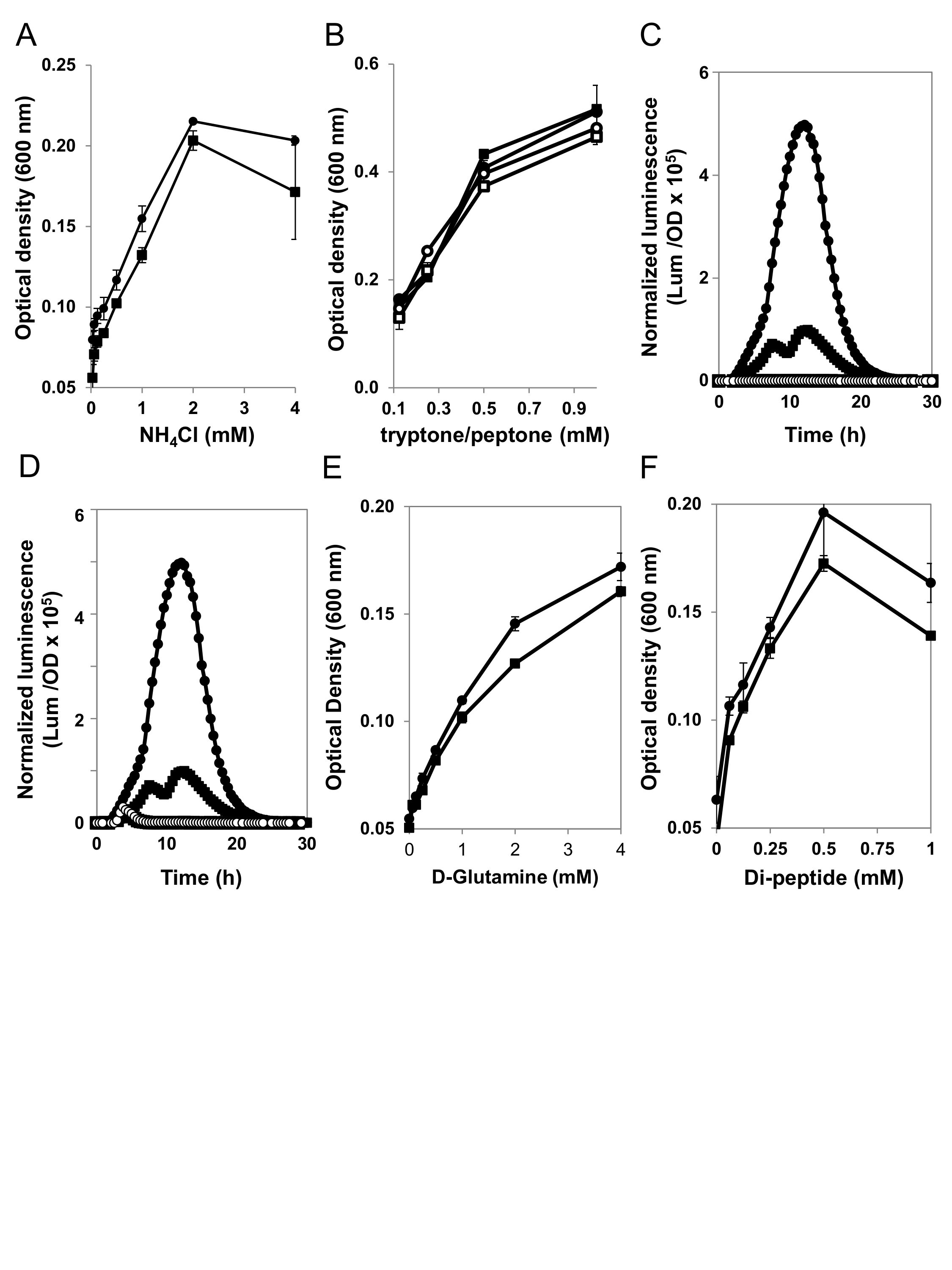

Supplement: S6 Fig — (A) Optical density (600 nm) measurements after 30 h of growth of WT L. monocytogenes (circles) or ΔglnPQ (squares) bacteria in MDM supplemented with the indicated concentrations of Ammonium chloride as the sole nitrogen source. Results are mean ±SD of 3 independent experiments, performed in triplicates. (B) Optical density (600 nm) measurements after 30 h of growth of WT L. monocytogenes (circles) or ΔglnPQ (squares) bacteria in MDM supplemented with the indicated concentrations of tryptone (full symbols) or peptone (open symbols) peptide digest as the sole nitrogen source. Results are mean ±SD of 3 independent experiments, performed in triplicates. (C) Time course measurements of normalized luminescence (Lum/OD) indicative of hly promoter activity in WT L. monocytogenes (circles), ΔglnPQ (squares) or glnQ-E164A (triangles) bacteria grown in MDM with 0.5 mM of L-glutamine (full symbols) or a peptone (open symbols) peptide digest. Shown are representative curves of 3 independent experiments, performed in triplicates. (D) Same as C only 0.5 mM of L-glutamine (full symbols) or a tryptone (open symbols) peptide digest. (E) Optical density (600 nm) measurements after 30 h of growth of WT L. monocytogenes (circles) or ΔglnPQ (squares) bacteria in MDM supplemented with the indicated concentrations of D-Glutamine as the sole nitrogen source. Results are mean ±SD of 3 independent experiments, performed in triplicates. (F) Same as E, only the indicated concentrations of an L-Gly-L-Gln di-peptide were used as the sole nitrogen source. (TIFF) [file ppat.1006161.s006.tiff]

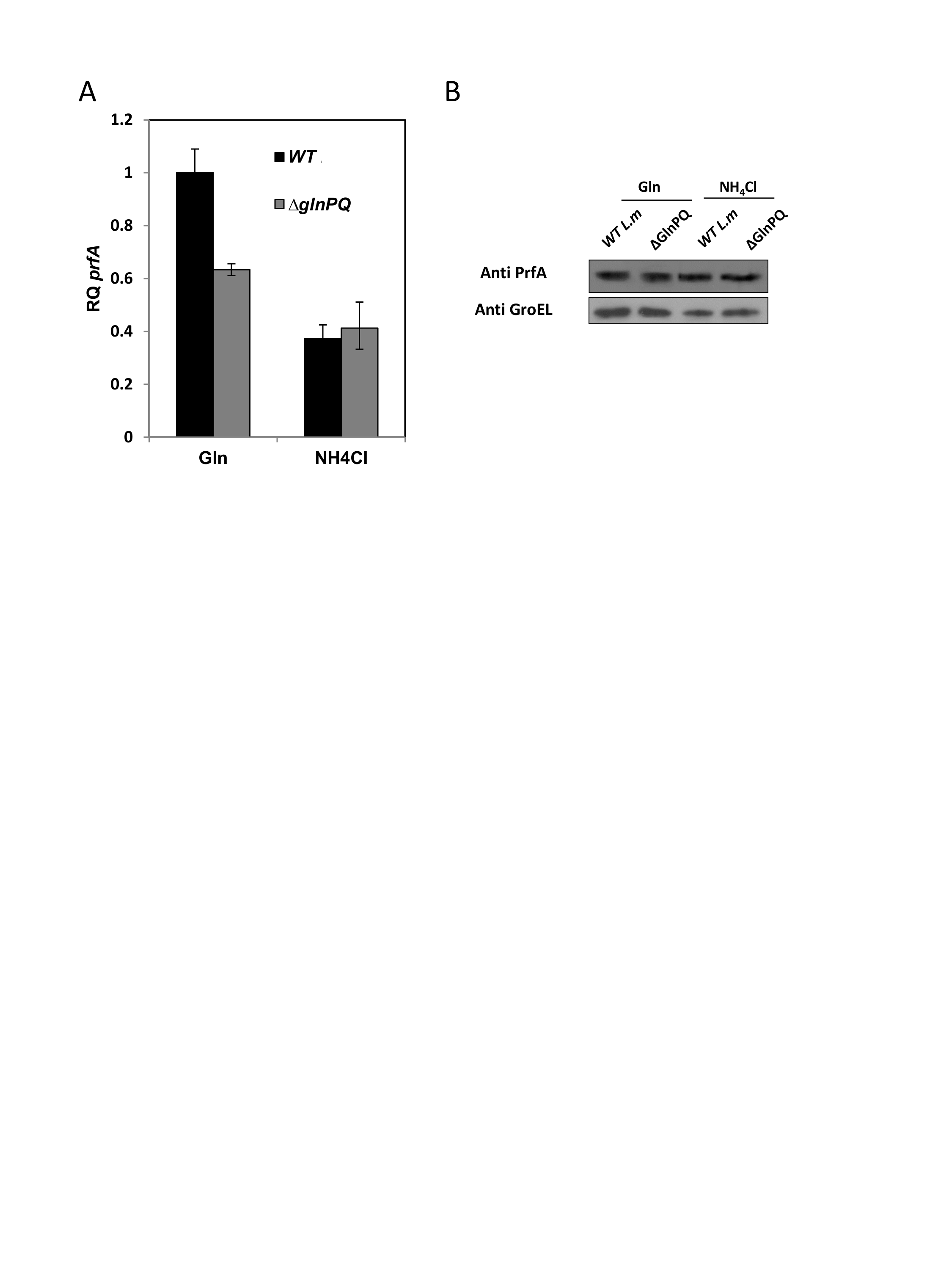

Supplement: S7 Fig — WT L. monocytogenes and ΔglnPQ bacteria were grown in the presence of 4 mM of L-glutamine or NH4Cl and prfA transcript levels were measured using real-time q-PCR analysis (A), while PrfA protein levels were detected by Western blot analysis using an anti-PrfA antibody (B). Transcription levels are presented as relative quantity (RQ), relative to levels in WT bacteria grown with L-glutamine. Results are mean ±SD of 3 independent experiments performed in triplicates. The Western blot shown is representative of three independent biological repeats. (TIFF) [file ppat.1006161.s007.tiff]

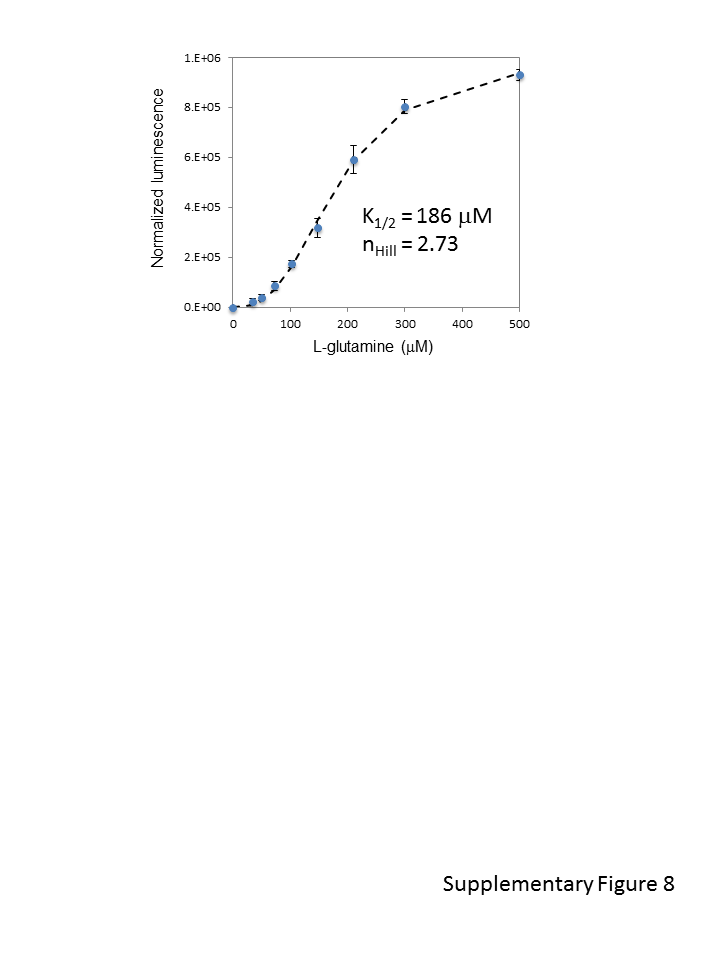

Supplement: S8 Fig — Shown is the peak luminescence measurments (blue diamonds) of WT bacteria grown in MDM supplemented with the indicated concentrations of L-glutamine. The dashed line is the fit of the data using the Hill equation. Also given are the K1/2 and the Hill coefficient nHill. (TIF) [file ppat.1006161.s008.TIF]
