# Supplementary material for: L-glutamine Induces Expression of Listeria monocytogenes Virulence Genes
Source: PLoS Pathog. 2017 Jan 23;13(1):e1006161. doi: 10.1371/journal.ppat.1006161 (PMC5289647; doi:10.1371/journal.ppat.1006161)
Supplement: S1 Table — (PDF) [file ppat.1006161.s009.pdf]

Supplementary Table 1 Strains used in this study

**Strain**	**Source**
L. monocytogenes 10403S	Daniel Portnoy Stock
L. monocytogenes 10403S Δ*glnPQ*	This study
L. monocytogenes 10403S Δ*glnPQ* E164A	This study
L. monocytogenes 10403S Δ*glnPQ* P*glnPQ*	This study
E. Coli SM10	R. Simon, U. Priefer A. Pühler (1983) A Broad Host Range Mobilization System for *In Vivo* Genetic Engineering: Transposon Mutagenesis in Gram Negative Bacteria *Nature Biotechnology* **1**, 784 - 791
