# Supplementary material for: L-glutamine Induces Expression of Listeria monocytogenes Virulence Genes
Source: PLoS Pathog. 2017 Jan 23;13(1):e1006161. doi: 10.1371/journal.ppat.1006161 (PMC5289647; doi:10.1371/journal.ppat.1006161)
Supplement: S2 Table — (PDF) [file ppat.1006161.s010.pdf]

Supplementary Table 2 Oligonucleotides used in this study

**Construction of strains**	
del-*LMRG_02270*-1 A	AAAATCTAGAAGGGACGAGCTACCCATATA
del-*LMRG_02270*-1 B	GTCATCTTGCTTTTCTGTTTATAACTTCATATTGTGTCTCCCTCTAT
del-*LMRG_02270*-1 C	ATAGAGGGAGGACACAATATGAAGTTATAAACGAAAAGCAAGATGAC
del-*LMRG_02270*-1 D	AAAACCCGGGCCATCTGGTTCAAGTTCTGG
glnPQ complementation F	TTTTGGTACCGCTTATATTGCATACGCAAAAATG
glnPQ complementation R	TTTTGAGCTCTGCTTTTGGAAATTCCAAAGTCATC
LMRG_02271 E164A A	AAAATACGTAGTACTTGGACATGGCTAATCATC
LMRG_02271 E164A B	GATCGAGGGCTGATGTAGGTGCATCGAACAACATAATGTCAGGATC
LMRG_02271 E164A C	GATCCTGACATTATGTTGTTCGATGCACCTACATCAGCCCTCGATC
LMRG_02271 E164A D	TTTTTACGTACTGGATGCTGTCGTAACAATTG
pPL2_*yfp* A	ATATGGGCCCGGGTTTCACTCTCCTTCTACATT
pPL2_*yfp* B	ACCTCCTTAGCTAGCAATGGCCCCCTCCTT
pPL2_*yfp* C	AAGGAGGGGGCCATTGCTAGCTAAGGAGGT
pPL2_*yfp* D	AATTCGGCCGTTAAAGCTTTTTATACAGTTCGT

Supplementary Table 2 continued- Oligonucleotides used in this study

**SBP point mutation**	
SBP–R105A	TGGCATGTCTATTACCGACGAAGCTAAACAAAAATTTGATTTCAGCG
SBP–R105A - RC	CGCTGAAATCAAATTTTTGTTTAGCTTCGTCGGTAATAGACATGCCA
**Macrophage gene expression RT-qPCR**	
IFN-β F	CCA AGA AAG GAC GAA CAT TCG
IFN-β R	CCG CCC TGT AGG TGA GGT T
GAPDH F	TTGTGGAAGGGCTCATGACC
GAPDH R	TCTTCTGGGTGGCAGTGATG
IL-6 F	TTCCATCCAGTTGCCTTCTTG
IL-6 R	GAAGGCCGTGGTTGTCACC
**Bacterial gene expression RT-qPCR**	
rpoD F	GTGATCGGACGGTGAAGTTG
rpoD R	ATCGCACAAGAGCCAGTTTC
hly F	AAACACGCGGATGAAATCGA
hly R	TAACCTTTTCTTGGCGGCAC
plcA F	ATGTCCACGTCATGTCTCCG
plcA R	GGATGTCCGCTCTACCTGAC
actA F	CGTCGTCGGATAGTGAGCTT
actA R	CCACAACTGACTCTTTCGCC
plcB F	GCAACGGAAGACATGGTAGC
plcB R	TAGTCCGCTTTCGCCCTTTT
